# An integrated integral projection model (IPM^2^ ) to disentangle size‐structured harvest and natural mortality

**DOI:** 10.1111/1365-2656.70176

**Published:** 2025-11-11

**Authors:** Abigail G. Keller, Benjamin R. Goldstein, Leah Skare, Perry de Valpine

**Affiliations:** ^1^ Department of Environment Science, Policy, and Management University of California, Berkeley Berkeley, California USA; ^2^ Department of Forestry and Environmental Resources North Carolina State University Raleigh North Carolina USA; ^3^ Northwest Straits Commission, Washington Department of Ecology Mount Vernon Washington USA

**Keywords:** European green crab, integral projection model, integrated population model, invasive species, size structure, state–space framework

## Abstract

1. Body size is one of the most important traits governing individual‐level demographic rates and modulating population‐level processes. Multiple size‐dependent demographic rates can simultaneously change population structure, so distinguishing their individual contributions to overall population dynamics remains a challenge.
2. Disentangling size‐dependent harvest rates from other demographic rates is critical for assessing the impact of removal on populations of invasive species. Inference about invasive populations can be difficult, however, as observations are often collected opportunistically as part of removal programs, rather than experimentally designed. Yet accurate inference is essential for understanding the feasibility of population suppression and optimising management decisions.
3. We develop an integrated integral projection model (IPM^2^) that leverages the strengths of the integrated population model and integral projection model to enable inference about complex, size‐structured demographic rates from imperfect observations. We apply the IPM^2^ in the context of invasive European green crab (*Carcinus maenas*), a species for which individual body size strongly regulates both the observation‐generating process and latent, population dynamics.
4. The IPM^2^ facilitates the distinct estimation of green crab size‐structured harvest and natural mortality rates, parameters for which no explicit data is collected and that are unidentifiable in component datasets of the integrated population model. The model represents how the green crab population changes over time, providing the first estimates of size‐structured abundance of this high‐priority species.
5. By forecasting the stable size distribution and equilibrium population size under varying removal efforts, we demonstrate that extremely high levels of removal effort can reduce the equilibrium green crab population size. Yet these high mortality rates also shift the stable size distribution and increase the equilibrium abundance of smaller crabs, since size‐selective removal alters intraspecific interactions. The ecological outcome of this shift in size structure will be variable, as green crab size modulates only some of its interactions with other species. These results highlight the value of the IPM^2^ framework for inferring complex population dynamics with information needs that outpace information in individual observational datasets, providing a path forward for accurate assessment of conservation programs.

## Graphical abstract

An IPM^2^—a combination of an integrated population model and integral projection model—creates a synergistic modelling framework useful for disentangling multiple size‐structured demographic rates and understanding the effectiveness of invasive European green crab removal efforts.

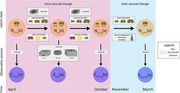

## 1 INTRODUCTION

Assessing the efficacy of invasive species management interventions requires understanding if and how removal actions change population abundance and dynamics. However, effective population suppression can be challenging to differentiate from natural biological variation, reflecting the perennial challenge of distinguishing harvest and natural mortality (Aanes et al., 2007). Often variability in harvest mortality is confounded with variability in natural mortality (Lewy & Nielsen, 2003), yet separating these fluctuations is crucial for understanding population processes and designing effective management strategies (Walters & Martell, 2004). Invasive species removal program success is highly variable (Prior et al., 2018), so reliably quantifying harvest rates is essential for evaluating intervention success and efficiently allocating limited management resources (Green & Grosholz, 2021).

This challenge of disentangling sources of mortality is amplified for species with complex, size‐structured demography. Body size can determine the strength of ecological interactions an individual experiences and can influence its key life‐history processes like growth and mortality (De Roos et al., 2003). The ontogenetic scaling of ecological performance with body size can ultimately drive patterns at the population level (Werner, 1994). Population dynamics can be shaped by competitive and predatory (cannibalistic) intraspecific interactions between different size cohorts (Claessen et al., 2004), predator–prey size‐dependent functional responses (Aljetlawi et al., 2004), variable reproductive capacity across size (Hixon et al., 2014) and size‐selective rates of harvest (Tu et al., 2018). Since these size‐structured rates and interactions simultaneously modulate population dynamics, unraveling their individual contributions can be difficult and require substantial amounts of well‐structured data.

This poses a particular challenge in the context of monitoring invasive species populations, where ecologists are often limited to removal sampling data (Udell et al., 2022), which are typically collected opportunistically as part of removal programs, rather than experimentally designed (Rogosch & Olden, 2021; Tiberti et al., 2021). Consequently, these data fail to meet the strict assumptions of models commonly used to estimate abundance with animals removed successively from multiple sites for closed populations (Dorazio et al., 2005). These removal observations are often not collected systematically across time and space and can be associated with large measurement error that can mask biological signals (Auger‐Méthé et al., 2016; Katsanevakis et al., 2012; Sibert et al., 2003).

Despite the unique statistical inference challenges associated with invasive species removal data, the costs of under‐ or overestimating removal harvest rates can be high. Ineffective invasive population suppression plans waste human and economic resources and can degrade the confidence of people and stakeholders involved in removal (Tiberti et al., 2021). As invasive species management becomes more ambitious in scope and scale, population control can be controversial, stimulating conflicts among people and debates about achievability and efficiency (Crowley et al., 2017). These undesirable social outcomes underscore the need for rigorous assessments of the effectiveness of control programs.

Combining existing classes of models can be useful for distinguishing process from observation dynamics, facilitating parameter identifiability and enabling inference about complex, size‐structured demographic rates from imperfect observations. Two attractive frameworks are integral projection models and integrated population models. An integral projection model can be used to make population projections based on vital rates that vary continuously as a function of body size (Merow et al., 2014; Rees et al., 2014). In contrast to matrix population models that can produce artefacts from coarse, arbitrary size class divisions, the integral projection model enables modelling of more biologically realistic, smooth relationships between individual size and demographic performance (Ellner & Rees, 2006). Formulated in a state–space framework, the integral projection model can be used to model natural variation in ecological processes separately from observation error (Auger‐Méthé et al., 2021; White et al., 2016).

Integrated population models can enable inference when the information requirements of these complex models exceed the information available in a single, limited dataset (Besbeas et al., 2002). By incorporating information from multiple survey and demographic datasets in an integrated framework, integrated population models increase the precision of parameter estimates and facilitate the estimation of additional parameters that would otherwise not be identifiable (Abadi et al., 2010; Riecke et al., 2019). Coupling an integrated population model and an integral projection model into an integrated integral projection model (IPM^2^) offers a fruitful synergy for understanding complex biological dynamics. While the integral projection model provides a biologically realistic mathematical representation of size‐structured dynamics, the integrated population model can make inference possible via the combined information from multiple datasets. Importantly, the IPM^2^ can be used to understand how the relationship between individual size and demographic performance can play a role in population‐level processes (Plard et al., 2019).

An IPM^2^ will be useful for understanding population dynamics and removal effectiveness of invasive European green crab (*Carcinus maenas*). Listed as one of the world's 100 worst invaders (Lowe et al., 2000), the green crab has multiple documented modes of impact. The green crab is an ecosystem engineer; through its foraging activity, the green crab physically alters eelgrass habitat and reduces the density of this important coastal habitat‐forming plant species (Garbary et al., 2014; Howard et al., 2019). The green crab also predates upon and outcompetes other shellfish, including commercially valuable bivalves like soft‐shell clams and manila clams (Fisher et al., 2024; Grosholz et al., 2000). This damage is costly, as potential future shellfish harvest losses due to green crab predation on the U.S. West Coast are forecasted to exceed $45 million per year (Grosholz et al., 2011).

Despite these impacts, the effectiveness of removal is not well characterised, as the crab's population dynamics are highly non‐linear and are regulated by complex, size‐structured demography. Adult crabs exert direct control of recruitment, largely through strong negative adult–juvenile interactions like cannibalism (Grosholz et al., 2021; Romano & Zeng, 2017). Competition with native crabs is also size‐structured, with larger crabs maintaining a competitive advantage for space and resources (Jensen et al., 2002; McDonald et al., 2001). Importantly, these individual, size‐structured interactions modulate population‐level processes; a field experiment in California, USA found a 30‐fold, single‐year increase in total green crab abundance in response to a removal program that selected for large adults (Grosholz et al., 2021). This high level of juvenile survival associated with low adult abundance, often referred to as overcompensation or the ‘hydra effect’, underscores the importance of quantifying size‐structured population response to removal (Abrams, 2009). The green crab observation process is also size‐selective, further complicating distinction between simultaneous size‐structured processes. Baited traps used for removal do not catch all individuals with equal probability across size classes, with the larval stage and smaller green crab size classes completely unobserved (Jørgensen et al., 2009). Additionally, the observability of the population changes within a trapping season (April–October), as juvenile individuals that have settled from their larval stage in the spring grow into size classes capable of being caught in traps by the fall. Quantifying the relationship between removal effort and size‐structured removal rates will be necessary for assessing the feasibility and impact of removal, understanding long‐term dynamics and optimising management actions and decisions for this high‐priority species.

**FIGURE 1:**
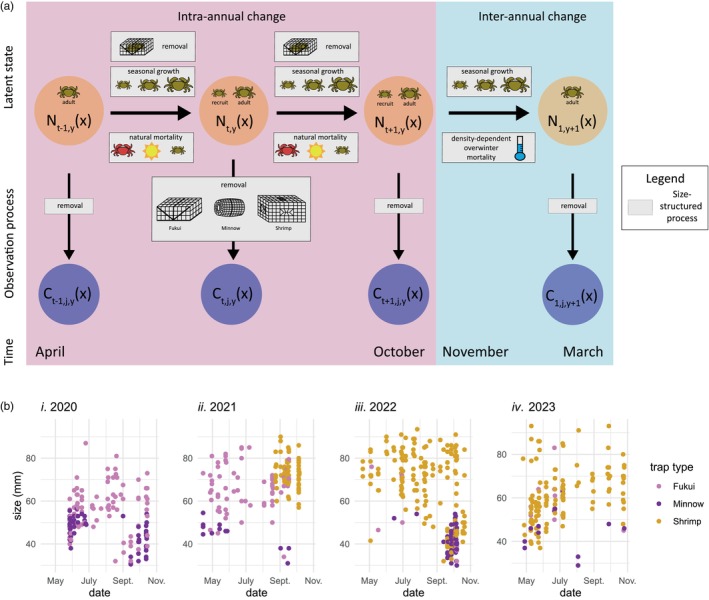
(a) Conceptual diagram of state–space population model, including the dependence structure in the latent process dynamics and observation process. Orange circles designate the population density of individuals of size x, during year y, at time t and are distinguished by dynamics within a year (intra‐annual change, April–October) and dynamics between years (inter‐annual change, November–March). Blue circles designate the count of removed crabs during time t, in trap j, in year y, of size x in the multi‐year time series dataset (D1). Grey boxes represent size‐structured demographic and observation processes. (b) Multi‐year time series data (D1) collected in Drayton Harbour from 2020 to 2023, highlighting the relationship between time and crab size (carapace width, mm). Each point corresponds to one captured crab, and colour corresponds to the type of trap used in capture.

**FIGURE 2:**
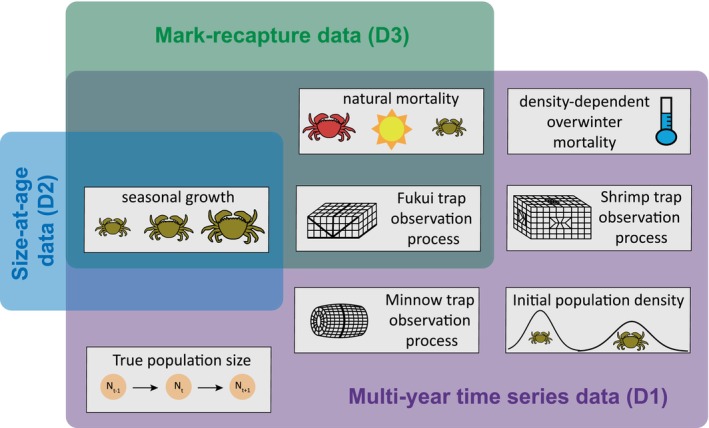
Overview of parameters informed by the three datasets in the integrated population model: Multi‐year time series data (D1) (Figure 1b), size‐at‐age data (D2) and mark‐recapture data (D3). Parameter categories correspond to categories designated in Table 1.

Here we develop an integrated integral projection model (IPM^2^) to quantify size‐structured harvest rates of invasive European green crab. As part of a state–space framework, process dynamics are described using an integral projection model, where the population structure changes over time through seasonal growth, natural mortality and removal, and observations are generated through the use of multiple size‐selective removal methods (Figure 1). Combining multiple datasets in an integrated framework allows for distinct inference about size‐structured harvest and natural mortality rates, both of which are ‘additional parameters’ for which no explicit data is collected. Three datasets contribute to different aspects of the model, with size‐at‐age data informing seasonal growth rates, mark‐recapture data primarily informing natural mortality and trap capture rates, and a multi‐year time series dataset merging all components and informing demographic and observation processes across multiple years (Figure 2). The IPM^2^ facilitates prediction of the stable size distribution and equilibrium abundance under different removal strategies, providing a framework for assessment of the effectiveness of invasive species control programs.

## 2 METHODS AND MATERIALS

### 2.1 Study system

While the European green crab (*Carcinus maenas*) has successfully colonised all continents except Antarctica (Yamada, 2001), our analysis focuses on the dynamics of green crab found in the Northeast Pacific along the west coast of North America, where the crab's range has been expanding over the last few decades. The first introduced green crab population in the Northeast Pacific was established in San Francisco Bay in the early 1990's. The crab's range has since expanded northwards through larval transport in the Davidson Current (Yamada et al., 2021); the green crab expanded into Oregon and Washington coastal estuaries in 1998 and into the Salish Sea in 2016 and 2017.

The European green crab is a resilient marine invader, able to withstand a wide range of environmental conditions and rapidly develop cold tolerance adaptations (Tepolt & Palumbi, 2020). Additionally, the green crab can recolonise quickly after removal, largely due to a mismatch in spatial scale between local removal programs and the crab's wide‐ranging dispersal and population processes (Keller, Counihan, et al., 2025). While adult crabs occupy sheltered bays and estuaries, the crab disperses during a larval phase in open marine waters that facilitates recolonisation of suppressed populations in neighbouring habitats (Yamada et al., 2021). Due to this spatial mismatch and rapid rate of recolonisation, some population control measures have had limited utility in sustained suppression over the longer term (Duncombe & Therriault, 2017; Tummon Flynn et al., 2024).

### 2.2 Demographic data

Population‐level inference is focused on size‐structured green crab abundance at Drayton Harbour in Washington, USA in the Salish Sea (Appendix 1.1, Figure A1.1). The primary dataset (D1) comprises a time series of counts of removed crabs collected in April–October from 2020 to 2023 and is structured by body size (Figure 1b). Multiple trap types (Fukui, Minnow and Shrimp) with different size‐selective removal rates were used (Figure 1b), and traps were baited and left to ‘soak’ in intertidal and subtidal habitat for 24–48 h. As Drayton Harbour is a protected bay, nearly enclosed by a land spit, we assume a closed population for adults and no movement of non‐larval crabs in and out of the study site.

Inference was supplemented by two additional datasets, size‐at‐age data (D2) and mark‐recapture data (D3). Size‐at‐age data (D2) were collected from crab removal observations associated with range expansions in northeastern Pacific estuaries documented by Yamada et al. (2021). Since the colonising cohorts of crabs following expansion events were relatively easy to identify as they aged over time, Yamada et al. (2021) assigned a year class to captured crabs based on the location of collection, assumed expansion event, date of capture, carapace width, sex and moult conditions (Yamada et al., 2021). Size‐structured mark‐recapture data (D3) were included to inform trap capture rate parameters of Fukui traps. These data were collected as part of a mark‐recapture experiment in July–November 2024 at Roche Cove on Vancouver Island in British Columbia, Canada. Here, crabs were captured and marked, released and recaptured over several time points.

More information on all three datasets can be found in Appendix 1.

### 2.3 Model description

We start by detailing the overall state–space IPM^2^ model, including the process and observation sub‐models (Figure 1). Description of all model parameters can be found in Table 1.

**TABLE 1:** Notation and biological meaning of data, latent states and parameters.

Symbol	Description	Category
*Demographic parameters*		
$\mu^{A}$	Log‐mean adult size in millimetres at t=1 and y=1	Init
$\sigma^{A}$	Log‐standard deviation of adult size in millimetres at t=1 and y=1	Init
$\mu^{R}$	Mean recruit size in millimetres upon entry into the process model at t=6	Init
$\sigma_{R}$	Standard deviation of recruit size in millimetres upon entry into the process model at t=6	Init
$x_{\infty }$	Asymptotic average crab size (carapace width, in mm)	Growth
k	Exponential rate of approach to the asymptotic size	Growth
A	Parameter modulating the amplitude of seasonal growth oscillations	Growth
$d_{s}$	Time (fraction of a year) between t=0 (April) and the start of the convex portion of the sinusoidal growth oscillation (i.e. inflection point)	Growth
$\sigma_{G}$	Standard deviation of somatic growth	Growth
$d_{0}$	Theoretical age a crab is of size zero millimetres	Growth
$\sigma_{w}$	Log‐standard deviation of log‐normally distributed error term to account for heterogeneity in individual growth rates in the size‐at‐age data	Growth
$\sigma_{u}$	Standard deviation of normally distributed error term to account for non‐independence among size‐at‐age data collected in the same year	Growth
β	Intensity of size‐independent natural mortality (not in winter months)	N. mort
α	Scalar that modulates the intensity of size‐dependent natural mortality (not in winter months)	N. mort
$\alpha^{o}$	Intensity of overwinter density‐ and size‐dependent natural mortality	O. mort
$\sigma_{o}$	Standard deviation of year‐specific overwinter mortality random effect	O. mort
*Observation parameters*		
$h^{\max}$	Maximum harvest mortality hazard rate. Maximum rate is trap type‐specific, such that $h_{F}^{\max}$, $h_{M}^{\max}$ and $h_{S}^{\max}$ correspond to Fukui, Minnow and Shrimp traps, respectively	F Obs, M Obs, S Obs
$h^{k}$	Steepness of change from the minimum to the maximum hazard rate for trap types with a logistic size‐selective function, such that $h_{F}^{k}$ and $h_{S}^{k}$ correspond to Fukui and Shrimp traps, respectively	F obs, S obs
$h^{0}$	Midpoint of change from the minimum to the maximum hazard rate for trap types with a logistic size‐selective function, such that $h_{F}^{0}$ and $h_{S}^{0}$ correspond to Fukui and Shrimp traps, respectively	F obs, S obs
$h_{M}^{A}$	Crab size associated with maximum hazard rate with Minnow traps	M obs
$h_{M}^{\sigma }$	Width parameter in the Minnow size‐selectivity hazard rate function	M obs
ρ	Parameter that describing overdispersion in the Dirichlet‐multinomial mixture distribution	F obs, M Obs, S Obs
*Population‐level quantities*		
$N_{t,y}\left(x\right)$	Population density function of individuals of size x, during year y, at time t	Pop. size
${\tilde{N}}_{t,y}\left(x_{i}\right)$	Discretised population density of individuals of size $x_{i}$, during year y, at time t	Pop. size
$N_{t,y}$	Total population abundance across all sizes, during year y, at time t	Pop. size
$\lambda^{A}$	Adult abundance at the first time period, t=1, during the first year, y=1	Pop. size
$\lambda_{y}^{R}$	Recruit abundance in year y	Pop. size
$\mu^{\lambda }$	Log‐mean recruit abundance	Pop. size
$\sigma^{\lambda }$	Log‐standard deviation of recruit abundance	Pop. size
*Observational data*		
$C_{t,y}^{T}\left(x_{i}\right)$	Total count of removed crabs across all traps during time t, in year y, of discrete size $x_{i}$ in the multi‐year time series dataset (D1)	—
$C_{t,j,y}\left(x_{i}\right)$	Count of removed crabs in time t, in trap j, in year y, of discrete size $x_{i}$ in the multi‐year time series dataset (D1)	—
$O_{t,y}$	Number of observations (traps) in time t, in year y in the multi‐year time series dataset (D1)	—
$W_{z,u}\left(a\right)$	Size of crab z, of age a, during year u in the size‐at‐age dataset (D2)	—
$m_{t}^{\mathrm{mc}}\left(x_{i}\right)$	Number of marked and released crabs of discrete size $x_{i}$ at time t in the mark‐recapture dataset (D3)	—
$r_{t}^{\mathrm{mc}}\left(x_{i}\right)$	Number of recaptured crabs of discrete size $x_{i}$ at time t in the mark‐recapture dataset (D3)	—
$O_{t}^{\mathrm{mc}}$	Number of observations (Fukui traps) at time t in the mark‐recapture dataset (D3)	—

*Note*: Category refers to the parameter categories designated in Figure 2: (1) Init is the size structure of initial population density and annual recruits, (2) Growth is seasonal growth, (3) N. mort is size‐dependent and size‐independent natural mortality in non‐winter months, (4) O. mort is size‐ and density‐dependent overwinter mortality, (5) F obs, M obs and S obs correspond to the size‐selective observation process for Fukui, Minnow and Shrimp traps, respectively, and (6) Pop. size corresponds to the true population size in the state–space model (Figure 1).

#### 2.3.1 Process model

The process model describes how the population at Drayton Harbour changes through time due to growth, natural mortality, removal and annual recruitment (Figure 1a). The following process equations describe the integral projection model that uses a kernel to project the population forward in time based on seasonal size‐dependent growth and size‐dependent natural survival (Rees et al., 2014). Over the winter between years (November–March; Figure 1a), size‐dependent natural survival in the kernel becomes density‐dependent. We then describe the annual recruitment process and how the time series is initialised with the first population density. These equations detail how the population changes during the trapping season (intra‐annual change; April–October) and during the winter season (inter‐annual change; November–March) (Figure 1a). The model tracks the state of the population in terms of its distribution of carapace sizes, $N_{t,y}\left(x\right)$, which is the density function of individuals of size x during year y at time t. We use a two‐week time interval between t and t+1, and we incorporate environmental stochasticity as growth variation at each time step, as well as demographic stochasticity in density‐dependent overwinter mortality.

#### 2.3.2 Integral projection model

The population density by size, $N_{t,y}\left(x\right)$, is projected forward in time using an integral projection model. The integral projection model is discrete in time and continuous over size x, and for estimation, the abundance of individuals is discretised using a small size interval (Δx=5 mm). The total population size, $N_{t,y}$, is $\int_{x\in \Omega }N_{t,y}\left(x\right)\mathrm{dx}$, where Ω represents all biologically feasible sizes (0–110 mm).

A kernel, $K_{t}\left(x^{\prime },x\right)$, describes the probability density of moving from size x to size $x^{\prime }$ at time t. $N_{t+1,y}\left(x\right)$ is therefore a function of $N_{t,y}\left(x\right)$, $K_{t}\left(x^{\prime },x\right)$, the total (across traps) size density of crabs removed in each time period, $C_{t,y}^{T}\left(x\right)$ and recruit abundance, $R\left(x\right)$. Recruits annually enter the model at one time point, $t_{R}$, where I is a binary variable indicating whether $t=t_{R}$:

(1) $$N_{t+1,y}\left(x^{\prime }\right)=\int_{x\prime \in \Omega }K_{t}\left(x^{\prime },x\right)\left(N_{t,y}\left(x\right)-C_{t,y}^{T}\left(x\right)\right)\mathrm{dx}+R\left(x\right)I_{t=t_{R}}$$

The kernel is defined as the product of a growth kernel, $G_{t}\left(x^{\prime },x\right)$ and size‐dependent natural survival, $S\left(x\right)$:

(2) $$K_{t}\left(x^{\prime },x\right)=G_{t}\left(x^{\prime },x\right)\times S\left(x\right)$$

#### 2.3.3 Seasonal growth

Like many ectotherms, green crab growth is strongly seasonal, with the growth rate peaking in the summer due to seasonal variation in temperature, light and food availability (Contreras et al., 2003; García‐Berthou et al., 2012). We therefore use a seasonal growth model that modifies the traditional von Bertalanffy growth model to incorporate seasonal growth oscillations by including a sine function with a period of 1 year (Beverton & Holt, 2012; Somers, 1988). The below growth equation describes growth from the theoretical age of a crab when it is of size 0, $d_{0}$, to the expected size of a crab at age a, $\tilde{W\left(a\right)}$. Here, $x_{\infty }$ is the asymptotic average size, k is a measure of the exponential rate of approach to the asymptotic size, A modulates the amplitude of the growth oscillations, and $d_{s}$ is the time (fraction of a year) between t=0 (April) and the start of the convex portion of the sinusoidal growth oscillation (i.e. inflection point) (García‐Berthou et al., 2012). Note that the $d_{0}$ parameter is not of biological interest and is instead a modelling artefact commonly used as an offset for age, as most fitted models do not pass through the origin (García‐Berthou et al., 2012; Schnute & Fournier, 1980).

(3) $$\tilde{W\left(a\right)}=x_{\infty }\left(1-\exp\left(-k\left(a-d_{0}\right)-s\left(a\right)+s\left(d_{0}\right)\right)\right)$$

(4) $$s\left(a\right)=\frac{\mathrm{Ak}}{2\pi }\sin\left(2\pi \left(a-d_{s}\right)\right)$$

However, since the age of the captured crabs in the multi‐year time series dataset (D1) is unknown, we use an equation to describe incremental growth from size x to $x^{\prime }$ derived from Equation 3 (White et al., 2016). Here, $d_{t}$ and $d_{t+1}$ are the year fraction associated with t and t+1 (i.e. $d_{t}=0$ for Julian day 91, $d_{t}=0.5$ for Julian day 274), and $\mu_{x,t+1}^{G}$ is the mean size at t+1, given x at t.

(5) $$\mu_{x,t+1}^{G}=x+\left(x_{\infty }-x\right)(1-\exp\left(-\mathrm{k\Delta t}-s\left(d_{t+1}\right)+s\left(d_{t}\right))\right)$$

Using the large sample approximation, we distribute individuals following a normal distribution across sizes. The growth kernel, $G_{t}\left(x^{\prime },x\right)$, is therefore described as:

(6) $$G_{t}\left(x^{\prime },x\right)=q\times \phi \left(x^{\prime };\mu_{x,t+1}^{G},\sigma_{G}^{2}\right);x,x^{\prime }\in \Omega$$

where $\sigma_{G}$ is the standard deviation in the growth rate, and ϕ is the normal probability density function. The constant, *q*, is used to normalise the kernel due to the truncated range of size, such that $\int_{x\prime \in \Omega }G_{t}\left(x^{\prime },x\right){\mathrm{dx}}^{\prime }=1$.

#### 2.3.4 Natural mortality

The rate of natural mortality decreases with size, as smaller crabs have lower intra‐ and inter‐specific competitive abilities and are more susceptible to predation and cannibalism (Grosholz et al., 2021; Maszczyk & Brzezinski, 2018). Natural survival during the non‐winter season, S, is described as:

(7) $$S\left(x\right)=\exp\left(-\mathrm{\Delta t}\left(\beta +\frac{\alpha }{x^{2}}\right)\right)$$

where β is the unitless intensity of size‐independent natural mortality, and α (which has units of length) is a scalar that modulates the intensity of size‐dependent natural mortality (Carlson et al., 2010).

#### 2.3.5 Overwinter growth and density‐dependent mortality

To transition from the last time point, $t_{\max}$, in year y to the first time point in year y+1, the population density experiences seasonal growth and density‐ and size‐dependent overwinter mortality.

The size distribution after seasonal growth and before overwinter mortality, $M_{y}\left(x\right)$, is a function of the population density at the onset of winter $N_{t_{\max},y}\left(x\right)$, the size density of removed crabs at the onset of winter $C_{t_{\max},y}^{T}\left(x\right)$ and the overwinter growth kernel $G^{o}\left(x^{\prime },x\right)$. Here, $G^{o}\left(x^{\prime },x\right)$ follows the same incremental seasonal growth described in Equation 5, except $d_{t}$ and $d_{t+1}$ are replaced with the year fraction associated with the onset of winter, $d_{t_{\max}}=0.53$ and the first time point in the following year, $d_{1}=0$.

(8) $$M_{y}\left(x\right)=\int_{x\prime \in \Omega }G^{o}\left(x^{\prime },x\right)\left(N_{t_{\max},y}\left(x\right)-C_{t_{\max},y}^{T}\left(x\right)\right)\mathrm{dx}$$

Due to thermal stress and starvation, the intensity of overwinter mortality is likely stronger than other times of the year and plays an important role in population regulation through density‐dependent control on population size (Henderson et al., 1988). Overwinter mortality is also size‐selective; smaller animals tend to have lower energy reserves than larger animals and use reserves more rapidly due to the allometry of metabolic rate (Hurst, 2007). Probability of overwinter survival, $S^{o}$, is therefore modelled as a density–size interaction, such that the intensity of size‐dependent overwinter mortality increases at higher population densities.

(9) $$S_{y}^{o}\left(x,N_{t_{\max},y}\right)=\exp\left(-\left(\frac{\alpha^{o}\times N_{t_{\max},y}}{x^{2}}+\epsilon_{y}\right)\right)$$

Process error enters as a year‐specific overwinter mortality random effect, with standard deviation, $\sigma_{o}$.

(10) $$\epsilon_{y}∼\text{Normal}\left(0,\sigma_{o}\right)$$

To implement model estimation, we discretise the continuous size distribution x to discrete size intervals $x_{i}$. The abundance of crabs in each size interval surviving the winter, ${\tilde{N}}_{1,y+1}\left(x_{i}\right)$, is drawn from a binomial distribution, where the number of trials is the rounded abundance of crabs after seasonal growth in each size interval, ${\tilde{M}}_{y}\left(x_{i}\right)$, and the probability of success is the probability of overwinter survival, $S_{y}^{o}\left(x_{i}\right)$. More information about the discretisation can be found in the Observation Model section below.

(11) $${\tilde{N}}_{1,y+1}\left(x_{i}\right)∼\text{Binomial}\left({\tilde{M}}_{y}\left(x_{i}\right),S_{y}^{o}\left(x_{i},N_{t_{\max},y}\right)\right)$$

Since density dependence during overwinter mortality plays an important role in long‐term population dynamics, we performed a model selection procedure to test a variety of formulations for Equation 9 (further described in the Model Fitting section below).

#### 2.3.6 Initial population density and annual recruitment

To initiate the process model time series, we estimate the size distribution and density of adults at the beginning of the first year. We also estimate the size‐structured abundance of recruits that enter the process model each year.

The first size distribution, $N_{1,1}\left(x\right)$, is defined in terms of the abundance of adults in the first time period during the first year, $\lambda^{A}$, the log‐mean initial adult size in millimetres, $\mu^{A}$ and the log‐standard deviation of initial adult size in millimetres, $\sigma^{A}$. Since the first year of multi‐year time series data (D1) in 2020, y=1, was at the start of green crab establishment in Drayton Harbour, we expected the size distribution to be dominated by age‐one crabs with a few age‐two crabs representing the first cohort of colonising crabs. We therefore assumed a log‐normal initial density to allow for a unimodal size distribution with a longer right tail. Here, $\phi_{L}$ represents the log‐normal probability density function.

(12) $$N_{1,1}\left(x\right)=\phi_{L}\left(x;\mu^{A},\sigma^{A}\right)\times \lambda^{A}$$

Ovigerous females spawn in August–December (Klassen & Locke, 2007), and these planktonic larvae exit estuarine habitat to develop in high salinity coastal waters alongside larvae produced by neighbouring habitats. Advection then brings larvae back into the estuary during recruitment (Young & Elliott, 2019). Recruitment is therefore modelled as an annual event with open demography, where the annual abundance of recruits, $\lambda_{y}^{R}$, is independent of adult abundance and follows a log‐normal distribution.

(13) $$\lambda_{y}^{R}∼\mathrm{Log}-\text{normal}\left(\mu^{\lambda },\sigma^{\lambda }\right)$$

The annual size distribution of recruits, $R_{y}\left(x\right)$, is defined in terms of the annual abundance of recruits, $\lambda_{y}^{R}$, the mean initial recruit size in millimetres, $\mu^{R}$ and the standard deviation of initial recruit size in millimetres, $\sigma_{R}$. ϕ represents the normal probability density function.

(14) $$R_{y}\left(x\right)=\phi \left(x;\mu^{R},\sigma_{R}^{2}\right)\times \lambda_{y}^{R}$$

Most crabs will settle from their planktonic larval stage in January to April (Yamada et al., 2005). Instead of estimating the time of planktonic settlement, we represent the recruits annually entering the population at a time point, $t_{R}$, (Equation 1) when (1) it can be assumed that the larvae have already settled as juveniles (i.e. mean initial recruit size, $\mu^{R}$, is greater than zero) and (2) well before the recruits grow into an observable size (Figure 1b). The recruits therefore enter the process model in mid‐May, corresponding to $t_{R}=6$.

#### 2.3.7 Observation model

The observation equations describe how the data were generated from the latent population quantities and demographic rates. A removal model was used to describe how the multi‐year time series data (D1) relates to the latent abundance, $N_{t,y}\left(x\right)$, at Drayton Harbour. Harvest mortality through trapping is described as a size‐selective hazard rate, which is informed by both the multi‐year time series data (D1) and mark‐recapture data (D3). The seasonal growth process was informed by the multi‐year time series data (D1) and size‐at‐age data (D2).

The process model equations in Section 2.3.1 describe the latent population dynamics at Drayton Harbor (D1), including the latent states, overwinter mortality, initial population density and recruitment process (Figure 2). Other process parameters, including the seasonal growth and natural mortality parameters, were informed by multiple datasets (Table 1, Figure 2). Below we also describe how D2 and D3 inform the shared parameters in the process equations (Figure 2).

To facilitate estimation of the IPM^2^ parameters, the latent abundance, $N_{t,y}\left(x\right)$, is discretised using a grid of values $x_{i},\ldots ,x_{m}$ that are regularly spaced such that Δx=5 mm and m=22. The discrete latent abundance, ${\tilde{N}}_{t,y}\left(x_{i}\right)$, is approximated in kernel transitions by the midpoint rule, where ${\tilde{N}}_{t,y}\left(x_{i}\right)=\int_{x_{i}-\frac{\mathrm{\Delta x}}{2}}^{x_{i}+\frac{\mathrm{\Delta x}}{2}}N_{t,y}\left(x\right)\mathrm{dx}$.

#### 2.3.8 Removal observation process (D1)

A removal model was used to describe the data‐generating process for the multi‐year time series data at Drayton Harbour (D1, Figure 1). Here, the removal count data, $C_{t,j,y}\left(x_{i}\right)$, represent the number of crabs of size $x_{i}$, caught at time t, during year y, in trap j, conditioned on the discrete size‐structured latent abundance, ${\tilde{N}}_{t,y}\left(x_{i}\right)$ (Kéry & Royle, 2015). Multiple traps were placed simultaneously in each time period, so we factor the probability of the removal count data in one time period as (1) a binomial distribution for the total number of removed crabs, $C_{t,y}^{T}\left(x_{i}\right)$ across all traps and (2) a Dirichlet‐multinomial distribution for the trap‐specific count, $C_{t,j,y}\left(x_{i}\right)$, describing which traps the crabs are caught in, given $C_{t,y}^{T}\left(x_{i}\right)$. Here, $C_{t,y}^{T}\left(x_{i}\right)=\sum_{j}C_{t,j,y}\left(x_{i}\right)$.

The total number of removed crabs, $C_{t,y}^{T}\left(x_{i}\right)$, follows a binomial distribution with number of trials equal to the discrete latent total abundance of crabs, ${\tilde{N}}_{t,y}\left(x_{i}\right)$, and total capture probability across all traps set during the same time period, $p_{t,y}\left(x_{i}\right)$.

(15) $$C_{t,y}^{T}\left(x_{i}\right)∼\text{Binomial}\left({\tilde{N}}_{t,y}\left(x_{i}\right),p_{t,y}\left(x_{i}\right)\right)$$

The vector size‐structured count of crabs in each trap j, $C_{t,y}\left(x_{i}\right)=\left(C_{t,1,y},\ldots ,C_{t,j,y}\right)$, follows a Dirichlet‐multinomial mixture distribution with overdispersion parameter, ρ and a vector of conditional probabilities of capture in trap j, ${p^{C}}_{t,y}\left(x_{i}\right)=\left(p_{t,1,y}^{C},\ldots ,p_{t,j,y}^{C}\right)$. Since the trap compositional data are overdispersed due to green crab aggregation and spatial behaviours, the Dirichlet allows for greater variance in the count data than predicted by a multinomial distribution (Thorson et al., 2017). More information on the Dirichlet‐multinomial mixture can be found in Appendix 2.2.

(16) $$C_{t,y}\left(x_{i}\right)∼\text{DirichletMultinomial}\left(C_{t,y}^{T}\left(x_{i}\right),{p^{C}}_{t,y}\left(x_{i}\right),\rho \right)$$

#### 2.3.9 Size‐selective hazard rates (D1 and D3)

Removal through trapping occurs in continuous time, described as a size‐selective hazard rate, $H\left(x\right)$, representing the instantaneous intensity of capture (Ergon et al., 2018). The shape and magnitude of this size‐selective hazard rate vary among the three trap types used for removal: Fukui, Minnow and Shrimp traps. Both the time series count data (D1) and mark‐recapture data (D3) inform the size‐selective probability of capture of Fukui traps, and only D1 informs the size‐selective probability of capture of Minnow and Shrimp traps (Figure 2).

Fukui and Shrimp traps capture larger crabs at higher rates than smaller crabs. The respective hazard rates of Fukui and Shrimp traps, $H_{F}\left(x\right)$ and $H_{S}\left(x\right)$, are modelled as a logistic function of crab size:

(17) $$H_{F}\left(x\right)=\frac{h_{F}^{\max}}{1+\exp\left\{-h_{F}^{k}\left(x-h_{F}^{0}\right)\right\}}$$

(18) $$H_{S}\left(x\right)=\frac{h_{S}^{\max}}{1+\exp\left\{-h_{S}^{k}\left(x-h_{S}^{0}\right)\right\}}$$

The Minnow trap mesh size is smaller than the maximum crab size, so this trap's size‐selective hazard rate, $H_{M}\left(x\right)$, follows a bell‐shaped curve (Jørgensen et al., 2009):

(19) $$H_{M}\left(x\right)=h_{M}^{\max}\times \exp\left(\frac{x-h_{M}^{A}}{2h_{M}^{\sigma }}\right)$$

Each baited trap, j, is placed in the habitat for a short (~24–48 h) time interval, $\Delta b_{t,j,y}$. The total capture probability, $p_{t,y}\left(x\right)$, is a function of the integrated hazard rate, summed across all traps set during the same time period, $O_{t,y}$.

(20) $$p_{t,y}\left(x\right)=1-\exp\left(-\sum_{j=1}^{O_{t,y}}H_{t,j,y}\left(x\right)\Delta b_{t,j,y}\right)$$

The conditional probability of capture, $p_{t,j,y}^{C}\left(x\right)$, is then $H_{t,j,y}\left(x\right)/\sum_{j=1}^{O_{t,y}}H_{t,j,y}\left(x\right)$.

#### 2.3.10 Size‐at‐age observation process (D2)

The size‐at‐age data, $W_{z,u}\left(a\right)$, indicate the carapace width of crab z at age a in year u. Since the age of the crabs in this dataset are recorded, unlike the time series dataset (D1), we used the unmodified seasonal growth equation that relates the theoretical age of the crab when it is of size 0, $d_{0}$, to the expected size of a crab at age a, $\tilde{W_{u}\left(a\right)}$ (Equation 3). A normally distributed error term, $\epsilon_{u}∼\text{Normal}\left(0,\sigma_{u}\right)$, is used to account for non‐independence among data collected in the same year, since growth rate is likely affected by water temperature, which varies from year to year.

(21) $$\tilde{W_{u}\left(a\right)}=x_{\infty }\left(1-\exp\left(-k\left(a-d_{0}\right)-s\left(a\right)+s\left(d_{0}\right)\right)\right)$$

To account for variation in growth rate, the observed size‐at‐age data, $W_{z,u}\left(a\right)$, follow a log‐normal distribution, with the expected carapace width, $\tilde{W_{u}\left(a\right)}$ and log‐standard deviation, $\sigma_{w}$.

(22) $$W_{z,u}\left(a\right)∼\mathrm{Log}-\text{normal}\left(\log(\tilde{W_{u}\left(a\right))},\sigma_{w}\right)$$

#### 2.3.11 Mark‐recapture observation process (D3)

The mark‐recapture data (D3) primarily informed the observation parameters that describe the size‐selective hazard rates of the Fukui trap type, $H_{F}\left(x\right)$ (Equation 17, Table 1, Figure 2), but also components of the growth and natural mortality kernel (Table 1; Figure 2). The mark‐recapture data consists of (1) $m_{t}^{\mathrm{mc}}\left(x_{i}\right)$, the count of marked and released crabs of discrete size $x_{i}$ for $t\in \left\{0\ldots t_{\max}^{\mathrm{mc}}-1\right\}$ and (2) $r_{t}^{\mathrm{mc}}\left(x_{i}\right)$, the count of recaptured and released crabs of discrete size $x_{i}$ for $t\in \left\{1\ldots t_{\max}^{\mathrm{mc}}\right\}$ (Appendix 1.3).

The count of marked and released crabs at t=0, $m_{0}^{\mathrm{mc}}\left(x_{i}\right)$, underwent seasonal growth and natural mortality to the first recapture time period, t=1, where $N_{1}^{\mathrm{mc}}\left(x_{i}\right)$ is the total marked crabs at t=1.

(23) $$N_{1}^{\mathrm{mc}}\left(x\prime_{i}\right)=\int_{x\in \Omega }K_{0}^{\mathrm{mc}}\left(x^{\prime },x\right)m_{0}^{\mathrm{mc}}\left(x_{i}\right)\mathrm{dx}$$

The total number of marked crabs, $N_{t}^{\mathrm{mc}}\left(x_{i}\right)$, in the remaining recapture time periods $t\in \left\{2\ldots t_{\max}^{\mathrm{mc}}-1\right\}$ undergo seasonal growth and natural mortality:

(24) $$N_{t+1}^{\mathrm{mc}}\left(x\prime_{i}\right)=\int_{x\in \Omega }K_{t}^{\mathrm{mc}}\left(x^{\prime },x\right)\left(N_{t}^{\mathrm{mc}}\left(x_{i}\right)+m_{t}^{\mathrm{mc}}\left(x_{i}\right)\right)\mathrm{dx}$$

The count of recaptured and released crabs, $r_{t}^{\mathrm{mc}}\left(x_{i}\right)$, are drawn from a binomial distribution where the size is the total number of marked crabs, $N_{t}^{\mathrm{mc}}\left(x_{i}\right)$ and probability, $p_{t}^{\mathrm{mc}}\left(x_{i}\right)$:

(25) $$r_{t}^{\mathrm{mc}}\left(x_{i}\right)∼\text{Binomial}\left(N_{t}^{\mathrm{mc}}\left(x_{i}\right),p_{t}^{\mathrm{mc}}\left(x_{i}\right)\right)$$

where $p^{\mathrm{mc}}\left(x_{i}\right)$ is the total probability of capture based on the total number of Fukui traps set, $O_{t}^{\mathrm{mc}}$, over the time period $\Delta b^{\mathrm{mc}}$:

(26) $$p_{t}^{\mathrm{mc}}\left(x\right)=1-\exp\left(-\sum_{j=1}^{O_{t}^{\mathrm{mc}}}H_{F,j}\left(x\right)\Delta b^{\mathrm{mc}}\right)$$

### 2.4 Model fitting

All three datasets were combined in a fully integrated model for joint estimation of model parameters. We fit the IPM^2^ in a Bayesian framework using NIMBLE v.1.2.1 (de Valpine et al., 2017). We used vague priors for all parameters, which are provided in Appendix 2.1. Parameters were estimated by running four Markov chain Monte Carlo (MCMC) chains of 100,000 iterations, thinned by a factor of 10. Of these 10,000 samples, 2000 were discarded as burn‐in. We used visual inspection of the MCMC chains and the Brooks and Gelman diagnostic $\hat{R}$ to assess model convergence, and we found that all parameters had an $\hat{R}\leq 1.1$ (Brooks & Gelman, 1998). All analyses were conducted in R version 4.4.1 (R Core Team, 2024). Code for the entire model is provided in Appendix 3, and generic, modular code that closely follows the model description is provided in Appendix 4. Posterior summaries, as well as convergence diagnostics and trace plots of model parameters can be found in Appendix 5.

To assess model performance and robustness, we conducted both a model selection and a model checking procedure (Conn et al., 2018). For model selection, we evaluated multiple functional forms of overwinter mortality using Watanabe–Akaike information criterion (WAIC). The inter‐annual population transitions (i.e. transition from year y to year y+1) are largely described by density‐dependent overwinter mortality. Since density dependence only enters the model during this process and is therefore likely influential for forecasting the stable size distribution, we compared multiple functional forms for size‐ and density‐dependent overwinter mortality and used the formulation with the lowest WAIC in the final analysis (Equation 9, Appendix 6.1).

To check the model, we calculated posterior predictive *p*‐values using deviance as an omnibus discrepancy function and proportion of zeros as a targeted discrepancy function to check for zero inflation of the count data (Appendix 6.2). We found that the model was an adequate representation of the data‐generating process, with a *p*‐value of 0.43 for the omnibus deviance discrepancy (Appendix 6.2; Figure A6.1). However, the *p*‐value for the proportion of zeros discrepancy function was 0.95, suggesting possible model misspecification (Appendix 6.2; Figure A6.2). These *p*‐values may be conservative; however, as Bayesian *p*‐values tend to be biased towards 0.5 (Conn et al., 2018).

### 2.5 Population forecasts

To evaluate the long‐term response of a green crab population to varying removal efforts, we approximated the stable size distribution and equilibrium abundance through stochastic forward simulations with posterior samples. Characterising the equilibrium abundance is most relevant in this context, as the green crab population is considered fully open and sustained local extinction is therefore unlikely. We randomly selected 1000 samples from the full posterior, and for each posterior sample, we generated an initial adult population size and projected the population forward 25 years, applying varying removal efforts for each set of 1000 simulations. These varying removal efforts included a total of 0, 112, 560 and 2800 annual Shrimp, Fukui or Minnow traps, applied evenly over the trapping season of 14 biweeks (12 total sets of 1000 simulations; four removal efforts × three trap types). Year‐varying recruit abundance and size‐ and density‐dependent overwinter mortality were drawn stochastically from process noise distributions each year in the forward simulations (Table 1). To reduce simulation noise between removal effort and trap type combinations, the same set of process noise stochastic draws for each posterior sample was consistent across the 12 simulation sets.

For each simulation, the size‐structured abundance at the end each of year after overwinter mortality, $N_{1,y+1}\left(x\right)$, was recorded (Figure 1a). Simulation outputs were summarised as the mean size‐structured abundance at the end of years 6–25, with the first 5 years treated as transient. We removed this transient period from the simulation summaries, since our objective was to characterise the long‐term response to different magnitudes of trapping, rather than the temporal dynamics as a population goes from untrapped to a new stable size distribution in response to trapping.

## 3 RESULTS

### 3.1 Estimating population‐level quantities

**FIGURE 3:**
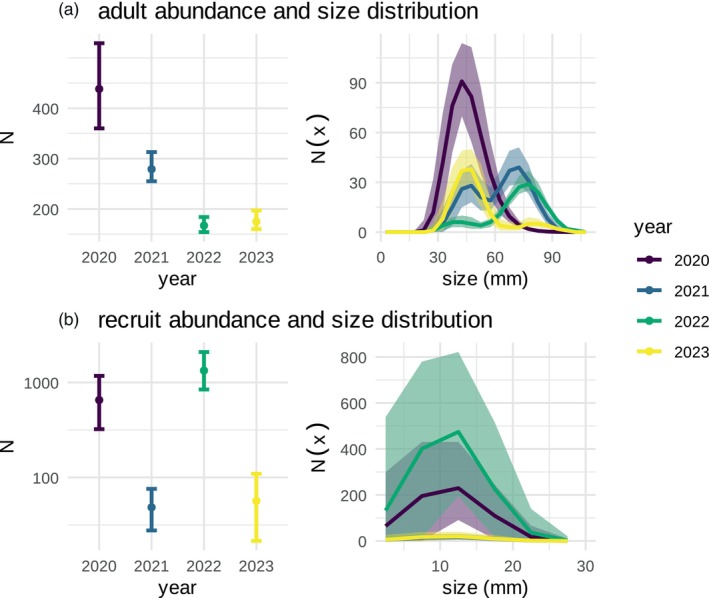
Total abundance and size distribution of (a) adults and (b) recruits in the fitted model. The left panels show the total abundance of crabs across all size classes, N, at the beginning of each year (i.e. $N_{1,y}$). The right panels show the size distribution, or the number of individuals in each size class, $N\left(x\right)$. Size corresponds to crab carapace width. Colours indicate the year, and error bars indicate the 95% credibility interval. Note that the left panel is the integral of the right panel.

The integrated integral projection model tracked the size‐structured European green crab abundance at Drayton Harbour (D1) throughout 2020–2023. Figure 3 shows the population density of adults and recruits at the beginning of each year. As the invasion progressed from 2020 to later years in 2021–2023, the size structure of adults shifted towards larger individuals (Figure 3a). This shift was particularly prominent in 2022 as a result of the low recruitment event in 2021, whereas the adult size distributions in 2021 and 2023 were more bimodal as a result of the large recruit age class in 2020 and 2022, respectively, that survived the winter but had not yet grown in size to match the sizes of crabs older than 1 year. This increase in median adult crab size after 2020 coincided with a decrease in overall adult crab abundance: total adult crab abundance decreased from 438 (358–524 95% CrI) individuals in 2020 to 280 (255–312 95% CrI), 168 (154–184 95% CrI) and 175 (159–195 95% CrI) in 2021–2023.

The abundance of recruits varied by multiple orders of magnitude across years, ranging from 694 (324–1150 95% CrI) and 1381 (841–2021 95% CrI) in the strong recruitment years of 2020 and 2022, to 50 (29–75 95% CrI) and 61 (39–108 95% CrI) in the weak recruitment years of 2021 and 2023 (Figure 3b). The mean size of recruits when they enter the model at t=6 each year (mid‐May) was 5.4 (1.0–9.8 95% CrI) millimetres (Figure 3b; Appendix 5). The credible intervals around recruit abundance and size are large (Figure 3b), since these recruits are unobserved until they grow into the observable size range in August–September (Figure 1b).

### 3.2 Distinguishing size‐structured natural and harvest mortality

By combining information in multiple datasets, the integrated population model allowed for estimation of additional parameters—size‐structured natural and harvest mortality—that were not identifiable with any one component dataset (Riecke et al., 2019).

**FIGURE 4:**
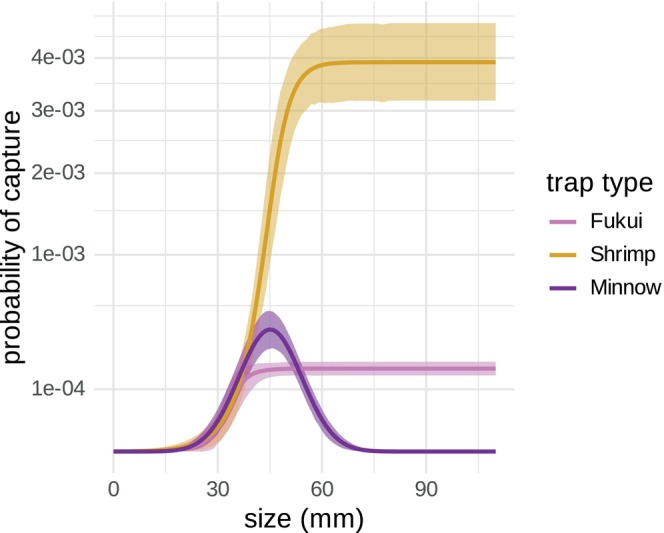
Size‐structured probability of capture, $p\left(x\right)$, in one trap over a 24‐h trapping period. Colours indicate the trap type. Note that the *y*‐axis is presented with a square root transformation.

Removal rates were estimated for three trap types—Fukui, Shrimp and Minnow—with different rates of removal and size selectivities (Figure 4). Overall, Shrimp traps removed crabs at the highest rate, and Minnow traps were only effective at removing crabs in the 30–60 mm size range. No trap effectively removed crabs smaller than 30 mm, consistent with the completely unobserved small crab portion of the population (Figure 1b).

**FIGURE 5:**
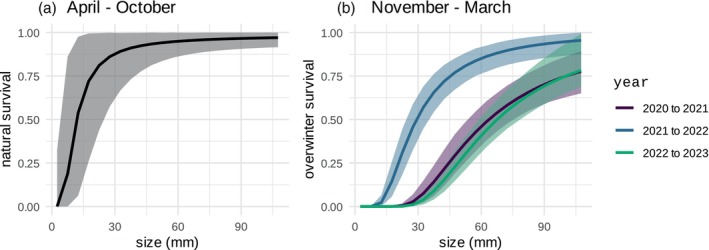
Size‐structured natural survival rate integrated over the (a). non‐winter season from April to October (Equation 7) and (b) year‐specific winter season from November to March (Equation 9). Colours indicate year, and error bars indicate the 95% credibility interval.

Natural survival rates over the five winter months were lower than natural survival rates during the seven other non‐winter periods of the year (Figure 5), consistent with the expectation that density dependence in overwinter mortality plays an important role in population regulation. Model comparison with WAIC demonstrated support for overwinter mortality as a function of the interaction between individual crab size and total population density (Appendix 6.1, Equation 9). Overwinter survival rates varied significantly from year to year, and correlated with total abundance and recruitment strength. Years with higher overall population density coincided with particularly low overwinter survival of small crabs (Figure 5b). In response to large recruitment events in 2020 and 2022 (Figure 3), over the winter following these years, survival rates dropped to 0.75 for crabs of the largest size and less than 0.25 for crabs of smaller size (Figure 5b). Conversely, survival rates were high over the winter between 2021 and 2022 in response to a small recruitment event in 2021 (Figure 3b) and an adult size structure biased towards larger crabs (Figure 3a).

### 3.3 Isolating growth in body size as a time‐ and size‐dependent process

Isolating the contribution of growth in body size in individuals to changes in population size structure helped facilitate inference of other size‐structured demographic rates. This growth rate was strongly seasonal and varied throughout the year, with growth rate peaking in the summer months and approaching zero in the winter months (Figure S1). The inflection point of the sinusoidal growth oscillation, $d_{s}$, indicates the time of year when body growth is most rapid. Since $d_{s}$ was 0.25 (0.21–0.28 95% CrI), and $d_{t}=0$ corresponds to April 1st (Julian day 91), the fastest rate of growth occurs around July 1st. The mean asymptotic crab size, $x_{\infty }$ was 80.7 mm (78.9–82.5 95% CrI) (Appendix 5). Growth was also variable; the standard deviation in growth rate, $\sigma_{G}$, was 2.8 mm (2.3–3.2 95% CrI) (Figure S1).

### 3.4 Population forecasts

**FIGURE 6:**
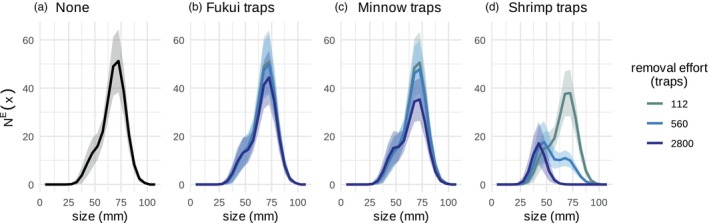
Population forecasts in response to varying removal efforts. Size distributions show the equilibrium crab abundance in each size class, $N^{E}\left(x\right)$, after overwinter mortality in April when (a) No traps, (b) only Fukui traps, (c) only Minnow traps or (d) only Shrimp traps were applied evenly over a trapping season of 14 biweeks. Solid line indicates the median size‐structured abundance across simulation replicates, and the shaded area indicates ±1 standard deviation across simulation replicates. Colours indicate total number of traps set over the trapping season.

Stochastic forward simulations with posterior samples were used to forecast the stable size distribution and equilibrium abundance under different levels of removal effort and subsequently different levels of harvest mortality (Figure 6). These simulations indicated that a low removal effort with Fukui and Minnow traps (Figure 6b,c) resulted in only marginal changes in the stable size distribution and equilibrium abundance relative to no removal effort (Figure 6a, Table S1). For example, the mean total equilibrium abundance, $N^{E}=\int_{x\in \Omega }N^{E}\left(x\right)\mathrm{dx}$, across simulation replicates with no removal was 270 crabs (Table S1). While the mean $N^{E}$ across simulation replicates with 2800 annual Fukui and Minnow traps was 239 and 210, respectively (Table S1). With a high removal effort of Shrimp traps, the total equilibrium abundance, $N^{E}$, decreases to 64 crabs, and large adult crabs can be completely removed from the population (Figure 6d, Table S1). However, this large crab removal merely shifts the stable size distribution towards smaller crabs; the equilibrium abundance of smaller crabs increases relative to no removal effort (Figure 6; Figure S2).

## 4 DISCUSSION

Our integrated integral projection model (IPM^2^)—a framework first described by Plard et al. (2019)—provides important insight for how body size can modulate many individual‐level demographic rates that interact to describe population‐level dynamics (Plard et al., 2019). The IPM^2^ produces an outcome greater than the sum of its parts: combining information in multiple datasets facilitates population inference for a species with complex demography with imperfect measurements. We are able to disentangle multiple size‐structured demographic rates that simultaneously change the size distribution over time (Carlson et al., 2010; Sogard, 1997), allowing detailed understanding of the individual contributions of each size‐structured demographic rate to overall population dynamics. These results also fill a significant knowledge gap for invasive European green crab (*Carcinus maenas*). While the species is highly studied due to its geographic ubiquity and management concern, this study is the first to quantify removal rates and size‐structured population abundance (Young & Elliott, 2019), providing a path forward for assessing feasibility of control and optimising management actions.

### 4.1 Disentangling size‐structured demographic rates

The IPM^2^ provides novel insights into previously uncharacterised size‐structured European green crab demographic rates, including size‐structured natural mortality, size‐ and density‐dependent overwinter mortality, recruitment processes, size‐structured growth rate and size‐selective harvest rates.

The model estimates unobserved quantities, most notably the abundance and size (carapace width) of recruits before they grow into observable size classes (Figures 1b and 3). Due to the size selectivity of most observation and removal methods, very little is known about the early stages of the green crab life cycle (Yamada et al., 2005), yet here, we used growth rates estimated by larger sizes to back‐calculate size and abundance of recruits before they are observed. However, the model may underestimate non‐overwinter natural mortality at smaller sizes, and therefore recruit abundance, because after settlement from the planktonic phase, recruits are unobserved for months and are likely dying naturally at very high rates (Figure 5a). Previous studies have reported extremely high densities of small juveniles (200–2000/m^2^) when counting individuals within quadrats in the intertidal zone at low tide where most juveniles are found (Breteler, 1976; Thiel & Dernedde, 1994). To better understand size‐dependent, non‐winter natural mortality, trap data could be integrated with measurements of small crabs made by other means, like visual or quadrat surveys.

Overwinter mortality is a major size‐selective process, as both energy reserves and metabolic rate scale with body size, such that larger individuals have higher energy reserves but lower metabolism and are therefore more resilient to starvation and physical extremes (Carlson et al., 2010; Sogard, 1997). Our results are consistent with this size‐dependent mortality pattern and provide support for the hypothesis that crab size and overall population density interact to modulate overwinter mortality (Appendix 6.1). Overwinter mortality varied significantly from year to year; years with higher overall population density coincided with particularly low overwinter survival of small crabs (Figure 5b). Overwinter mortality therefore likely plays an important role in regulating size‐structured green crab population dynamics (Henderson et al., 1988).

Recruitment varies by orders of magnitude from year to year, ranging from about 50 recruits in weak years to 1000–2000 in strong years (Figure 3b). The green crab has a long planktonic larval stage, living in open marine waters for months before advection back into the estuarine environment where they settle in the sub‐ and intertidal zone (Yamada, 2001). This inter‐annual variability in recruitment is consistent with varying oceanographic conditions; the survival and successful transport of larvae often coincide with warm winter water temperatures and oceanographic conditions like El Niño/Southern Oscillation (ENSO) and Pacific Decadal Oscillation (PDO) events (Yamada et al., 2021). For example, the first large green crab colonisation event at Drayton Harbor is linked to the 2018–2019 El Niño. These recruits would then grow throughout the year in 2019 to be associated with the high year‐one adult abundance observed in 2020 (Figure 3a). Though drawing from a small sample size of only 4 years, recruit abundance appears to be decoupled from adult abundance; for example, adult abundance was highest in 2020, yet recruit abundance was lowest in 2021 (Figure 3). This decoupling of recruit and adult abundances suggests that recruitment is likely driven by oceanographic conditions and regional population dynamics, rather than local adult abundance. However, a longer time series coupled with genomic information will be necessary for understanding the relative contribution of local and regional processes in population dynamics.

The model also estimates demographic rates that vary across time and size. Marine invertebrates are often subject to seasonal variation in environmental factors like photoperiod, food availability and temperature, resulting in elevated rates of growth, feeding and oxygen consumption in the summer season (Brockington & Clarke, 2001). Consistent with this expectation, we find that green crab growth is strongly seasonal, with growth rate peaking in summer months and approaching zero in the winter (Figure S1). Green crab growth rate is also size‐dependent, as the moulting rate is much higher at smaller crab sizes and slows down as crabs approach the mean asymptotic crab size (Yamada et al., 2005).

The IPM^2^ estimates absolute size‐selective capture rates for different trap types, allowing for the first estimates of size‐structured abundance of European green crab (Young & Elliott, 2019). These capture rate and abundance estimates mark an important advance for moving beyond catch per unit effort (CPUE) as the primary, yet imperfect, method for measuring population trends (Harley et al., 2001). These results also highlight the strong size selectivity of removal methods, since capture rates approach zero for crabs smaller than 30 millimetres (Figure 4). This size selectivity will have uncertain evolutionary consequences, yet resolving this uncertainty will be important for projecting long‐term population dynamics. Fishing‐induced life‐history evolution is frequently observed in harvested species (Enberg et al., 2012), and previous invasive species management programs have demonstrated rapid ecological and evolutionary changes in response to selective harvesting, including a shift towards earlier size at maturity and an overall slower growing phenotype (Evangelista et al., 2015). These phenotypic responses to removal programs can have strong effects on ecosystem recovery (Závorka et al., 2020), so understanding the role of green crab removal as a selection pressure will be important for managing this species capable of rapid adaptation (Tepolt & Palumbi, 2020).

### 4.2 Predicting the impact of removal on European green crab dynamics

These estimates of size‐structured demographic rates and size‐selective harvest rates will be essential for understanding the impact of removal on green crab dynamics and the feasibility of population suppression. Our model results show that harvest mortality associated with low levels of removal effort, especially with Fukui and Minnow traps, only marginally change the green crab equilibrium abundance and size structure, relative to doing nothing (Figure 6a–c; Table S1). These results highlight that low levels of removal effort can be useful for monitoring population trends but are insufficient for control and population suppression.

Even with extremely high levels of removal effort with the most effective trap type—Shrimp traps—control will mostly shift the stable size distribution of the population towards smaller crab sizes (Figure 6d; Figure S2). For example, if 2800 Shrimp traps are applied throughout the year, the resulting total equilibrium abundance, $N^{E}$, is 25% of the total equilibrium abundance if no traps were set (Table S1), yet the remaining crabs are all <60 mm (Figure 6d). These results are consistent with observations of decreased median carapace width in response to removal (de Rivera et al., 2007), and they support the prediction that though removal programs may achieve short‐term or local benefits, control is likely unable to sustainably suppress populations over larger temporal and spatial scales (Kanary et al., 2014; Keller, Counihan, et al., 2025; Tummon Flynn et al., 2024). In fact, strong removal pressure increases the equilibrium abundance of small crabs (Figure 6d, Figure S2). Removing adult crabs reduces the intraspecific regulation of recruits, resulting in higher abundance of smaller crabs relative to doing nothing. This increase in small crab abundance is similar to dynamics observed in an intensive control experiment by Grosholz et al. (2021), the first controlled experimental field demonstration of the ‘hydra effect’ (Grosholz et al., 2021). The ecological outcome of a removal‐induced shift in size structure will likely be location‐specific and depend upon the impacted species. While the competitive advantage between green crab and native crab species and the rate of predation upon Manila clams and adult Pacific oysters increases with crab size, crab size does not appear to affect the rate of eelgrass alteration and larval Pacific oyster consumption (Anaya et al., 2025; McDonald et al., 2001).

These results are dependent on the assumption of a completely open population, where the recruitment rate is independent of the local population size. This assumption, in general and in the case of Drayton Harbour in this study, is likely appropriate, as genomic analyses find high gene flow and low genetic structure across local green crab populations in the Northeast Pacific (Tepolt et al., 2009, 2022). However, these results may not be applicable in some circumstances where a local population is partially closed, often in unique oceanographic conditions that support strong larval retention within an estuary (Grosholz et al., 2021).

The population forecasts assume stationarity when predicting the equilibrium abundance and stable size distributions under varying removal efforts (Figure 6). While these forecasts allow recruitment to vary from year to year, the mean and variance of recruit abundance are constant over time. These forecasts would likely be different if recruitment is non‐stationary, either decreasing over time through intensive removal efforts across the region or at known source populations or increasing over time through increased colonisation pressure. Non‐stationarity in colonisation pressure is likely, as climate and oceanographic models project an increase in oceanographic events that support survival and transport of larvae (Cai et al., 2021; Du et al., 2024). Other demographic rates, like growth and natural mortality may also be non‐stationary. The integral projection model lends itself to understanding how changing environmental factors, like increased temperature, will affect individual size and overall population dynamics (Dahlgren & Ehrlén, 2011; Plard et al., 2019).

### 4.3 Reliability of parameter estimates in integrated population models

Integrated population models are useful for inferring parameters of interest in scenarios where information needs of complex models exceed information in individual datasets. Despite these benefits, often the reliability of parameter estimates remains underexamined. Simulation‐based approaches have shown that IPM parameters—particularly ‘additional’, previously unidentifiable parameters—are sensitive to violations of model assumptions (Riecke et al., 2019). In the green crab IPM^2^, management‐relevant model parameters, including size‐structured natural and harvest mortality, were not estimable from one data source but were indirectly estimable from multiple data sources and the assumed model structure. For example, the Roche Cove mark‐recapture dataset (D3) was critical for inferring trap hazard rates and abundance estimates at Drayton Harbour where the multi‐year time series data was collected (D1).

The integrated population model assumes that the same ecological process has generated these disparate datasets. Posterior predictive checks that compare the observed data to data generated by the model can be a valuable tool for evaluating violations of this assumption. For example, the deviance‐based Bayesian *p*‐value that tests the model's global lack of fit was 0.43 (Appendix 6.2). We also fit the model with an alternate mark‐recapture dataset collected at a different location, Seadrift Lagoon in California, USA (Appendix 6.3.1). The habitat at Drayton Harbour (D1) is more similar to Roche Cove (D3) than to Seadrift Lagoon, and these differences may impact inter‐ and intraspecific population processes (Appendix 6.3.1). The deviance‐based Bayesian *p*‐value calculated with the alternate Seadrift Lagoon mark‐recapture data was 0.71 (Appendix 6.3). Both *p*‐values suggest reasonable model fits, and although inclusion of the Seadrift Lagoon data does not qualitatively change the results, some parameter estimates do change moderately (Appendix 6.3). While posterior predictive checks may be useful for broadly understanding if multiple datasets reflect the same data‐generating process, selecting datasets for an integrated population model may be limited to judgments about habitat similarity.

The targeted posterior predictive check used to assess zero inflation in the count data at Drayton Harbour, $C{\left(x_{i}\right)}_{t,j,y}$, produced a Bayesian *p*‐value of 0.95. This result suggests that the model may inadequately account for processes that could generate overdispersion in the count data, like spatial heterogeneity in green crab distribution within Drayton Harbour or interactions between traps set simultaneously.

### 4.4 Embedding the IPM^2^ in a decision‐making framework

These results demonstrate that removal cannot eradicate an open population of European green crab (Figure 6). While removal can decrease the total equilibrium population size at a location like Drayton Harbor with relatively low colonisation pressure (Table S1), removal primarily shifts the stable size distribution towards smaller crabs (Figure 6d). In circumstances where invasive species eradication is infeasible, the management focus often moves towards functional eradication or suppression of the population below levels that cause unacceptable ecological effects (Green & Grosholz, 2021). The IPM^2^ green crab model may become valuable in a decision‐making framework to optimise removal actions and find the removal effort—and subsequently the stable size distribution and equilibrium abundance—that both minimises removal cost and impact on habitat, native species and natural resources.

Embedding this size‐structured model within a decision‐making framework, however, will require improved knowledge of size‐structured impacts and computational methods to optimise high‐dimensional decision problems. Green crab size often mediates its interactions between prey and competitors; other decapod species are preyed upon by green crab as juveniles but outcompete green crab as adults, and green crab often only predate upon bivalves of smaller size (Curtis et al., 2012; Grosholz, 2005; McDonald et al., 2001; Williams et al., 2009). Quantifying size‐ or biomass‐dependent impacts will be critical for optimising the allocation of removal resources in this size‐structured system. Additionally, this work highlights that green crab population dynamics cannot be represented in a one‐dimensional system (i.e. total abundance), since the size structure of the population plays an important role in long‐term dynamics. Techniques like stochastic dynamic programming can be used to optimise sequential decision problems (Marescot et al., 2013), yet due to the curse of dimensionality, these methods will be insufficient for optimising problems with large state and action spaces. The size‐structured green crab abundance changes within a single decision cycle, and multiple trap types have different size‐dependent removal rates and different monetary and logistical costs of use (Figure 3). Since the complexity of the decision problem scales non‐linearly with the size of the system, advanced computational methods like neural‐network‐based reinforcement learning or factored Markov decision processes will be needed to optimise management actions in this high‐dimensional decision problem (Lapeyrolerie et al., 2022; Nicol et al., 2015).

## AUTHOR CONTRIBUTIONS

Abigail G. Keller, Benjamin R. Goldstein and Perry de Valpine conceived the ideas and designed methodology; Leah Skare collected the data; Abigail G. Keller and Perry de Valpine analysed the data; Abigail G. Keller led the writing of the manuscript. All authors contributed critically to the drafts and gave final approval for publication.

## CONFLICT OF INTEREST STATEMENT

The authors have no conflict of interest to declare.

## DISCLAIMER

This report was prepared as an account of work sponsored by an agency of the United States Government. Neither the United States Government nor any agency thereof, nor any of their employees, makes any warranty, express or implied or assumes any legal liability or responsibility for the accuracy, completeness or usefulness of any information, apparatus, product or process disclosed or represents that its use would not infringe privately owned rights. Reference herein to any specific commercial product, process or service by trade name, trademark, manufacturer or otherwise does not necessarily constitute or imply its endorsement, recommendation or favouring by the United States Government or any agency thereof. The views and opinions of authors expressed herein do not necessarily state or reflect those of the United States Government or any agency thereof.

## Supporting information


**Appendix 1.** Description of data.

**Appendix 2.** Prior distributions and multinomial‐Dirichlet mixture.

**Appendix 3.** Model code.

**Appendix 4.** Generic model code.

**Appendix 5.** Posterior summaries and trace plots.

**Appendix 6.** Robustness Assessments.

**Figure S1.** Growth kernel, $G_{t}\left(x^{\prime },x\right)$ (Equation 6), that describes the probability of transitioning from size x to size $x^{\prime }$ at time t. Results show the probability of growing from x to $x^{\prime }$ over a 2‐week period (i.e. t to t+1) when t corresponds to April 1st, July 1st, October 1st and January 1st. Red dashed line shows the line where $x^{\prime }=x$. The growth kernel is calculated with the mean posterior estimates of growth parameters.
**Figure S2.** Population forecasts in response to varying removal efforts, relative to no removal. Size distributions show the ratio of the equilibrium crab abundance in each size class, $N^{E}\left(x\right)$, when no removal occurred ($N^{E}{\left(x\right)}_{\text{effort}=0}$) relative to a removal effort greater than zero ($N^{E}{\left(x\right)}_{\text{effort}>0}$). Ratios are calculated based on size‐structured abundance at the end of the year after overwinter mortality when 112 traps, 560 traps or 2800 traps were applied evenly over a trapping season of 14 biweeks with either *A*. Fukui traps, *B*. Minnow traps or *C*. Shrimp traps. A ratio of one means that the size‐structured abundance after no removal equals the size‐structured abundance after application of Z traps. A ratio less than one means that the application of Z traps removes decreases the size‐structured abundance, relative to no removal. A ratio greater than one means that the application of Z traps increases the size‐structured abundance, relative to no removal. Solid line indicates the median size‐structured abundance across simulation replicates, and the shaded area indicates ±1 standard deviation across simulation replicates.
**Table S1.** Total equilibrium abundance across all size classes, $N^{E}$ for population forecasts in response to varying removal efforts. Mean refers to the mean $N^{E}$ across all simulation replicates, and sd refers to the standard deviation of $N^{E}$ across all simulation replicates.

## Data Availability

All data and R code associated with this analysis can be found in the Zenodo archive: https://doi.org/10.5281/zenodo.16995754 (Keller, Goldstein, et al., 2025) and GitHub: https://github.com/abigailkeller/IPMsquared.
