# Supplementary material for: An integrated integral projection model (IPM2 ) to disentangle size‐structured harvest and natural mortality
Source: J Anim Ecol. 2025 Nov 11;95(1):157–74. doi: 10.1111/1365-2656.70176 (PMC12775557; doi:10.1111/1365-2656.70176)
Supplement: Supplementary file 1 — Appendix 1. Description of data. [file JANE-95-157-s003.pdf]

Appendix 1: Description of data

Appendix 1

The integrated population model provides a framework for synthesizing multiple demographic datasets to mutually inform shared parameters. In Appendix 1.1, we further describe the multi-year Drayton Harbor time series data (D1). In Appendix 1.2, we describe additional information about the size-at-age data (D2), and in Appendix 1.3, we further describe the mark-recapture data (D3).

Appendix 1.1

The multi-year time series data (D1) was collected in Drayton Harbor, an enclosed bay in the Salish Sea in Washington State, USA.

Figure A1.1: Bathymetric map of Drayton Harbor. Colors indicate depth and black line indicates coast line.

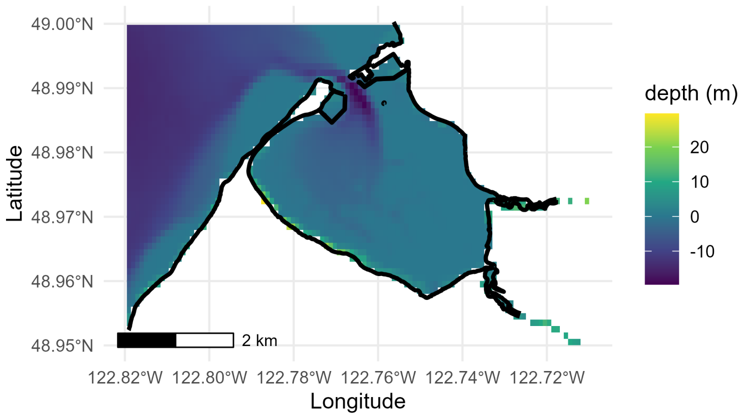

At Drayton Harbor, traps were baited and left to "soak" in intertidal and subtidal habitat for 24-48 hours. Trapped areas within Drayton Harbor include a large tideflat and the mouth of three creeks that feed into the bay (named "North", "Dakota Creek", "California Creek", and "Noname Creek"). After the "soak" time, traps were retrieved, and green crabs were counted and removed from the system. The carapace widths (CW) of green crabs were measured with vernier calipers between the tips of their fifth anterio-lateral spines to the nearest lowest mm. Multiple trap types (Fukui, Minnow, and Shrimp) with different size-selective removal rates were used. These traps have different mesh sizes and entrance openings: Fukui traps have 12 mm mesh with approximately 20 cm entrance openings; Gee-brand minnow traps have 6 mm mesh with approximately 5 cm entrances; Promar-brand 4-way Shrimp traps have 12.7 mm or 25.4 mm mesh size and approximately 10 cm entrance opening. These entrance and mesh sizes are approximate, however, as the traps can slightly change as they get used over time.

Data collected in Drayton Harbor data were subset to only include recruits (age < 1) that appear in a single cohort in the fall of each year (Figure 1B). This subset only removed three recruit individuals in 2022 from the dataset that were from an off-cycle recruitment cohort.

Below we describe the total removal effort and catch of European green crab (EGC, *C. maenas*) in Drayton Harbor (D1). While the removal count data is collected at many time points throughout the trapping season (April - October), we discretize the time intervals into biweekly periods in the model.

Table A1.1: Number of crabs caught and total removal effort (number of traps) during each biweek and year. Julian day signifies the first Julian day of the discretized biweek period. F, M, and S correspond to Fukui, Minnow, and Shrimp traps, respectively.

Julian day	year	F (catch)	F (effort)	M (catch)	M (effort)	S (catch)	S (effort)
137	2020	12	48	22	43	0	0
152	2020	16	67	10	62	0	0
167	2020	8	62	5	57	0	0
182	2020	4	100	0	96	0	0
198	2020	1	64	0	44	0	0
213	2020	12	61	0	60	0	0
229	2020	7	82	0	71	0	0
244	2020	8	97	4	87	0	0
259	2020	4	15	0	10	0	0
259	2020	15	57	23	52	0	0
91	2021	1	180	4	180	0	0
106	2021	7	168	1	168	0	0
121	2021	8	130	2	130	0	0
137	2021	9	207	2	205	0	0
152	2021	4	180	0	180	0	0
167	2021	6	198	0	198	0	0
182	2021	2	120	0	120	0	0
198	2021	0	123	0	123	0	0
213	2021	18	200	0	203	0	0
229	2021	1	251	1	251	12	27
244	2021	8	296	3	297	27	39
259	2021	0	0	0	60	23	24
106	2022	0	135	0	135	3	12
121	2022	2	195	0	195	13	17
137	2022	0	90	0	90	4	12
152	2022	0	127	1	125	4	16
167	2022	2	120	0	120	22	27
182	2022	0	0	0	0	4	6
198	2022	0	62	1	62	15	23
213	2022	0	207	0	207	20	71
229	2022	0	30	0	30	6	50
244	2022	0	0	0	0	5	26
259	2022	0	0	2	26	23	25
259	2022	7	27	29	118	37	50
290	2022	0	0	0	0	6	12
305	2022	8	30	16	30	16	10
106	2023	0	120	2	120	3	20
121	2023	2	163	1	163	20	31
137	2023	2	95	2	95	23	34
152	2023	0	0	0	0	12	26
167	2023	5	60	2	60	8	28
182	2023	0	80	2	80	18	14
198	2023	0	60	0	60	2	26
213	2023	0	43	2	45	2	22
229	2023	0	72	0	149	6	31
244	2023	0	15	0	45	0	18
259	2023	0	30	1	105	10	30
259	2023	0	0	0	0	0	6

Julian day	year	F (catch)	F (effort)	M (catch)	M (effort)	S (catch)	S (effort)
290	2023	1	45	1	90	14	112

Appendix 1.2

Size-at-age data (D2) were collected from crab removal observations associated with range expansions in northeastern Pacific estuaries documented by Yamada et al. 2021. Since the colonizing cohorts of crabs following expansion events were relatively easy to identify as they age over time, Yamada et al. 2021 assigned a year class to captured crabs based on the location of collection, assumed expansion event, date of capture, carapace width, sex, and molt conditions (Yamada et al. 2021).

For example, El Niño events, with their characteristic warm water temperatures and enhanced northward transport by the Davidson Current, are especially favorable to the survival and transport of larvae hundreds of kilometers from source populations. The 1997 to 1998 El Niño resulted in the colonization of embayments in Oregon, coastal Washington, and on the west coast of Vancouver Island from California, and the El Niño of 2015 to 2016 and the Pacific Warm Blob were linked to range expansion into the Salish Sea. More local range expansions in other areas also occurred during non-El Niño years.

Yamada et al. 2021 report an estimated year class for captured crabs, which corresponds to the first year of growth after settlement from its planktonic larval stage. We use this estimated year class and the reported crab capture date from this dataset to estimate the age of captured individuals. We assume planktonic settlement in March. For example, for a crab captured in September 2000 with estimated year class of 1998 would be assigned an age of 2.5.

Table A1.2: Number of size-at-age records during each year and waterbody in the northeastern Pacific.

Year	Waterbody	n
1995	Humboldt Bay	2
1997	Coos Bay	2
1998	Yaquina Bay	1
1999	Barkley Sound	2
1999	Price Bay	1
2000	Clayoquot Sound	1
2000	Nootka Sound	1
2001	Little Espinosa Inlet	3
2007	Klaskino Inlet	1
2011	Gale Passage	2
2015	Kildidt Inlet	2
2016	Higgins Passage	1
2016	Westcott Bay	1
2017	Becher Bay	3
2017	Padilla Bay	1
2017	Whidbey Island	2
2018	Port Townshend Bay	1
2018	Scow Bay	1
2018	Westcott Bay	2
2018	Whidbey Island	1
2018	Witty's Lagoon	1
2019	Bellingham	3
2019	Boundary Bay	3
2019	Chuckanut Bay	1
2019	Esquimalt Lagoon	2
2019	Lummi Bay	2
2019	Salt Spring Island	4
2019	Samish Bay	1
2019	Sequim Bay	3
2019	Westcott Bay	1
2019	Witty's Lagoon	2

Year	Waterbody	n
2020	Chuckanut Bay	1
2020	Daajing Giids	1

Appendix 1.3

The mark-recapture data (D3) were collected as part of a mark-recapture experiment in July through November 2024 at Roche Cove on Vancouver Island in British Columbia, Canada.

Green crab were trapped using baited Fukui traps, with trap lines consisting of six traps placed 10 meters apart. Lines were set approximately 10 m apart and set both parallel and perpendicular to the shore line to create an equal grid across the site, and traps "soaked" for about 24 hours before being checked. Captured crabs were measured, sexed, and color and health determined before marking. All crab were tagged using small sequentially numbered disk tags, with the edges trimmed down to size, glued to the hind end of the carapace using Crazy Glue, with accelerant sprayed lightly on to set. Crab were held in submerged sealed Fukui traps for 24-48 hours and, prior to release, the tags were individually checked for retention. Crab were evenly dispersed throughout the trapping location to ensure equal re-distribution across the site.

The experiment took place over four time periods, $t \in \{0...3\}$. Marking events took place at $t \in \{0...2\}$, where crabs were marked and released back into the environment. Recapture events took place at $t \in \{1...3\}$, and recaptured crabs at $t \in \{1, 2\}$ were released back into the environment (Figure A1.2).

Figure A1.2: Conceptual diagram of mark-recapture schema. The number of crabs marked at time t of size x is $m_t^{\mathrm{mc}}(x)$, the number of crabs recaptured at time t of size x is $r_t^{\mathrm{mc}}(x)$, and the total number of crabs marked at time t of size x is $N_t^{\mathrm{mc}}(x)$.

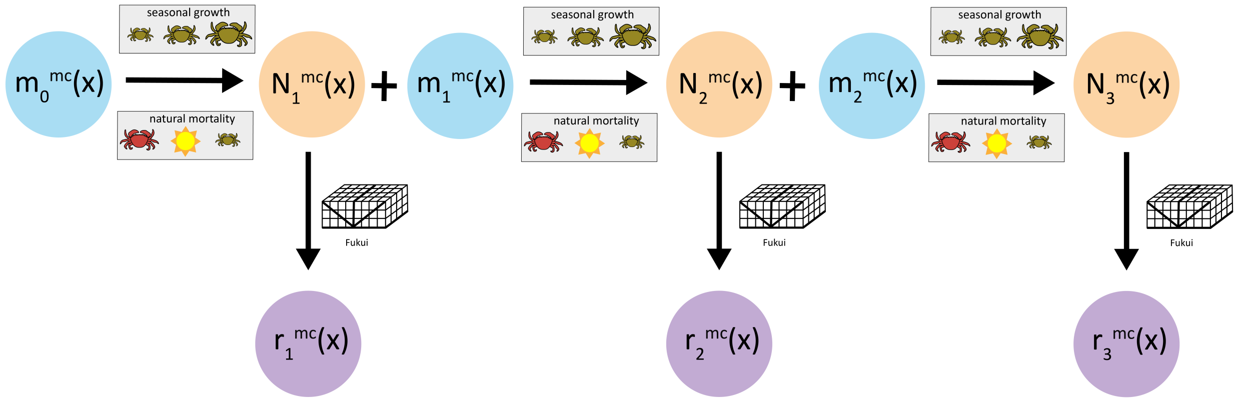

Across the four time periods, 234 total traps were set, 1676 crabs were marked, and 88 crabs were recaptured (Table A1.3).

Table A1.3: Total number of Fukui traps set, crabs marked (across all sizes), and crabs recaptured (across all sizes) for each of the four mark-recapture time periods.

t	Julian day	Total traps set	Total marked	Total recaptured
0	197	36	590	NA
1	213	54	558	23
2	244	54	528	37
3	305	90	NA	28

References

Yamada, Sylvia Behrens, Graham E Gillespie, Richard E Thomson, and Tammy C Norgard. 2021. "Ocean Indicators Predict Range Expansion of an Introduced Species: Invasion History of the European Green Crab Carcinus Maenas on the North American Pacific Coast." *Journal of Shellfish Research* 40 (2): 399–413.
