# Supplementary material for: An integrated integral projection model (IPM2 ) to disentangle size‐structured harvest and natural mortality
Source: J Anim Ecol. 2025 Nov 11;95(1):157–74. doi: 10.1111/1365-2656.70176 (PMC12775557; doi:10.1111/1365-2656.70176)
Supplement: Supplementary file 2 — Appendix 2. Prior distributions and multinomial‐Dirichlet mixture. [file JANE-95-157-s007.pdf]

# Appendix 2: Prior distributions and multinomial-Dirichlet mixture

## Appendix 2.1

The following vague prior distributions were used in the model. Parameter descriptions associated with parameter symbols can be found in Table 1 of the main text:

Trap size selectivity parameters:

$$h_M^{\max} \sim \text{Uniform}(0, 0.1)$$

$$h_M^A \sim \text{Uniform}(35, 60)$$

$$h_M^\sigma \sim \text{Uniform}(3, 10)$$

$$h_F^{\max} \sim \text{Uniform}(0, 0.1)$$

$$h_F^k \sim \text{Uniform}(0.1, 1.5)$$

$$h_F^0 \sim \text{Uniform}(30, 100)$$

$$h_S^{\max} \sim \text{Uniform}(0, 0.1)$$

$$h_S^k \sim \text{Uniform}(0.1, 1.5)$$

$$h_S^0 \sim \text{Uniform}(30, 100)$$

Natural mortality parameters:

$$\beta \sim \text{Uniform}(0, 10000)$$

$$\alpha \sim \text{Uniform}(0, 10000)$$

Overwinter mortality parameters:

$$\alpha^o \sim \text{Uniform}(0, 50)$$

$$\sigma_o \sim \text{Uniform}(0, 1000)$$

Seasonal growth parameters:

$$k \sim \text{Uniform}(0, 2)$$

$$A \sim \text{Uniform}(0, 4)$$

$$d_s \sim \text{Uniform}(0, 1)$$

$$d_0 \sim \text{Uniform}(-10, 10)$$

$$\sigma_w \sim \text{Uniform}(0, 100)$$

$$\sigma_u \sim \text{Uniform}(0, 100)$$

$$x_\infty \sim \text{Uniform}(70, 140)$$

$$\sigma_G \sim \text{Uniform}(0.01, 4)$$

Overdispersion observation process (see Appendix 2.2):

$$\rho \sim \text{Beta}(1, 1)$$

Initial population density and annual recruitment:

$$\mu^A \sim \text{Uniform}(3.25, 4.5)$$

$$\sigma^A \sim \text{Uniform}(0.1, 1)$$

$$\mu^R \sim \text{Uniform}(1, 25)$$

$$\sigma_R \sim \text{Uniform}(0.01, 20)$$

$$\mu^\lambda \sim \text{Uniform}(-50, 50)$$

$$\sigma^\lambda \sim \text{Uniform}(0, 10000)$$

$$\lambda^A \sim \text{Uniform}(1, 1000000)$$

## Appendix 2.2

Similarly to how a beta-binomial replaces a single probability with a Beta distribution of probabilities among binomial draws to account for overdispersion, the Dirichlet-multinomial mixture replaces a single vector of probabilities (that sum to one) with a Dirichlet distribution of such vectors to account for overdispersion in counts among traps.

The parameter $\alpha^D$ amount of overdispersion in the Dirichlet-multinomial mixture distribution and generates the conditional probability of capture, $p^C_{t,j,y}(x)$, across individual traps.

The parameter $\alpha^D$ is linked to the sampled parameter, $\rho$:

$$\alpha^D = p^C_{t,j,y}(x) \times p^D$$

$$p^D = \frac{1-\rho}{\rho}$$
