# Supplementary material for: An integrated integral projection model (IPM2 ) to disentangle size‐structured harvest and natural mortality
Source: J Anim Ecol. 2025 Nov 11;95(1):157–74. doi: 10.1111/1365-2656.70176 (PMC12775557; doi:10.1111/1365-2656.70176)
Supplement: Supplementary file 3 — Appendix 3. Model code. [file JANE-95-157-s005.pdf]

Appendix 3: Model code

This appendix contains the model code, written and implemented with the software `nimble` and implemented in parallel with the `parallel` package. See Appendix 4 for more generic, modular model code that closely follows the model description in the main text without the extra data structures required to account for the heterogeneous data used to fit the model.

First data will be read in:

```
library(nimble)
library(MCMCvis)
library(parallel)
library(expm)

# read in time series data
C <- readRDS("data/model_data/counts.rds")
C_T <- readRDS("data/model_data/n_cap.rds")

# read in time series constants
index <- readRDS("data/model_data/index.rds")
D <- readRDS("data/model_data/index_frac.rds")
totalt <- readRDS("data/model_data/totalt.rds")
totalo <- readRDS("data/model_data/totalo.rds")
soak_days <- readRDS("data/model_data/soak_days.rds")
m_index <- readRDS("data/model_data/m_index.rds")
f_index <- readRDS("data/model_data/f_index.rds")
s_index <- readRDS("data/model_data/s_index.rds")
recruit_intro <- readRDS("data/model_data/recruit_intro.rds")
additionalt <- readRDS("data/model_data/additionalt.rds")

# read in mark-recapture data
marked_rc <- readRDS("data/model_data/roche_mc_mark.rds")
catch_rc <- readRDS("data/model_data/roche_mc_catch.rds")

# read in mark-recapture constants - Roche Cove
D_mc_rc <- readRDS("data/model_data/mc_index_rc.rds")
totalo_mc_rc <- readRDS("data/model_data/roche_mc_totalo.rds")$n
soak_days_mc_rc <- readRDS("data/model_data/soak_days_mc_rc.rds")
f_index_mc_rc <- readRDS("data/model_data/f_index_mc_rc.rds")
s_index_mc_rc <- readRDS("data/model_data/s_index_mc_rc.rds")
m_index_mc_rc <- readRDS("data/model_data/m_index_mc_rc.rds")


# read in IPM constants
b <- readRDS("data/model_data/b.rds")
x <- readRDS("data/model_data/x.rds")

# read in growth data
growth_data <- readRDS("data/model_data/growth_data.rds")
```

Then the model code will be specified:

```
# Write model code
model_code <- nimbleCode({

  #################
  #################
  # Process model #
  #################
  #################

  ##############################
  # Integral projection model #
  ##############################

  # project to first observed time period - year 2-4
  for (y in 2:n_year) {
    N[1, y, 1:n_size] <- get_kernel(xinf, gk, sigma_G, A,
                                    ds, 0, D[1, y], n_size, pi,
                                    x[1:n_size], lower[1:n_size],
                                    upper[1:n_size], S[1, y, 1:n_size]) %*%
      N_overwinter[y - 1, 1:n_size]

    ## natural survival
    S[1, y, 1:n_size] <- survival(alpha, beta, x[1:n_size], 0, D[1, y])
  }

  # intra-annual change
  for (y in 1:n_year) {
    for (t in 1:(totalt[y]-1)) {

      # Equations 1 - 2
      ## project
      N[t + 1, y, 1:n_size] <- get_kernel(xinf, gk, sigma_G,
                                          A, ds, D[t, y],
                                          D[t + 1, y], n_size, pi,
                                          x[1:n_size], lower[1:n_size],
                                          upper[1:n_size],
                                          S[t + 1, y, 1:n_size])  %*%
        (N[t, y, 1:n_size] - C_T[t, y, 1:n_size]) +
        recruit_intro[t, y] * R[y, 1:n_size] # introduce recruits, t = 6

      # Equation 7
      ## natural survival
      S[t + 1, y, 1:n_size] <- survival(alpha, beta, x[1:n_size], D[t, y],
                                        D[t + 1, y])

    }
  }

  # project all years to the same intra-annual end point by
  # applying the growth kernel and natural mortality
```

```
for (y in 1:n_year_short) {
  for (t in additionalt[year_short[y], 1]:additionalt[year_short[y], 2]) {

    # Equations 1 - 2
    ## project
    N[t + 1, year_short[y], 1:n_size] <- get_kernel(xinf, gk, sigma_G,
                                                A, ds,
                                                D[t, year_short[y]],
                                                D[t + 1, year_short[y]],
                                                n_size, pi,
                                                x[1:n_size],
                                                lower[1:n_size],
                                                upper[1:n_size],
                                                S[t + 1, year_short[y],
                                                  1:n_size]) %*%
      N[t, year_short[y], 1:n_size]

    S[t + 1, year_short[y], 1:n_size] <- survival(alpha, beta, x[1:n_size],
                                              D[t, year_short[y]],
                                              D[t + 1, year_short[y]])
  }
}


## inter-annual change
# project to new year with seasonal growth
for (y in 1:(n_year - 1)) {
  wgrowth_N[y, 1:n_size] <- get_kernel(xinf, gk, sigma_G, A, ds,
                                  max_D, 1,
                                  n_size, pi, x[1:n_size],
                                  lower[1:n_size], upper[1:n_size],
                                  ones[1:n_size]) %*%
    N[additionalt[y, 2] + 1, y, 1:n_size]

  # total density after seasonal growth
  wgrowth_N_sum[y] <- sum(wgrowth_N[y, 1:n_size])

  # Equation 9
  ## size- and density-dependent overwinter survival
  S_o[y, 1:n_size] <- overwinter_survival(alpha_o,
                                     wgrowth_N_sum[y],
                                     x[1:n_size], eps_y[y])

  # Equation 10
  ## year-specific overwinter mortality random effect
  eps_y[y] ~ dnorm(0, sd = sigma_o)

  # Equation 11
  # size- and density-dependent overwinter mortality
  for (k in 1:n_size) {
    N_overwinter[y, k] ~ dbinom(size = round(wgrowth_N[y, k]),
                             prob = S_o[y, k])
  }
```

```r
}

#####################################################
# Initial population density and annual recruitment #
#####################################################

# Equation 12
## get initial size-structured abundance of adults - year 1
N_init[1:n_size] <- get_init_adult(log_mu_A, sigma_A,
                                   lower[1:n_size], upper[1:n_size], lambda_A)

# project to first observed time period - year 1
N[1, 1, 1:n_size] <- get_kernel(xinf, gk, sigma_G, A,
                                ds, 0, D[1, 1], n_size, pi,
                                x[1:n_size], lower[1:n_size],
                                upper[1:n_size], S[1, 1, 1:n_size]) %*%
  N_init[1:n_size]

## natural survival
S[1, 1, 1:n_size] <- survival(alpha, beta, x[1:n_size], 0, D[1, 1])

# Equation 13
## annual abundance of recruits
for (m in 1:n_year) {
  lambda_R[m] ~ dlnorm(mu_lambda, sdlog = sigma_lambda)
}

# Equation 14
## annual size distribution of recruits
R[1:n_year, 1:n_size] <- get_init_recruits(mu_R, sigma_R, lower[1:n_size],
                                           upper[1:n_size],
                                           lambda_R[1:n_year], n_year,
                                           n_size)


#####################
#####################
# Observation model #
#####################
#####################

for (y in 1:n_year) {
  for (t in 1:totalt[y]) {
    for (k in 1:n_size) {

      # Equation 15
      ## binomial distribution with total crabs removed, C_T
      C_T[t, y, k] ~ dbinom(size = round(N[t, y, k]), prob = p[t, y, k])

      # Equation 16
      ## dirichlet-multinomial mixture, conditional probability of capture
      alpha_D[t, 1:totalo[t, y], y, k] <- p_C[t, 1:totalo[t, y],
                                               y, k] * n_p_dir
```

```
      C[t, 1:totalo[t, y],
          y, k] ~ ddirchmulti(alpha = alpha_D[t, 1:totalo[t, y], y, k],
                              size = C_T[t, y, k])
    }

    # Equations 17 - 19
    ## calculate hazard rate
    hazard[t, 1:totalo[t, y], y, 1:n_size] <- calc_hazard(
      totalo[t, y], n_size, h_F_max, h_F_k, h_F_0, h_S_max, h_S_k, h_S_0,
      h_M_max, h_M_A, h_M_sigma, f_index[t, 1:totalo[t, y], y],
      s_index[t, 1:totalo[t, y], y], m_index[t, 1:totalo[t, y], y],
      soak_days[t, 1:totalo[t, y], y], x[1:n_size]
    )

    # Equation 20
    ## total capture probability
    p[t, y, 1:n_size] <- calc_prob(totalo[t, y], n_size,
                                   hazard[t, 1:totalo[t, y], y, 1:n_size])

    # mean conditional probability of capture
    p_C[t, 1:totalo[t, y],
        y, 1:n_size] <- calc_cond_prob(totalo[t, y], n_size,
                                       hazard[t, 1:totalo[t, y],
                                              y, 1:n_size])

  }
}

###############################
###############################
# Integrated population model #
###############################
###############################

################
# Growth model #
################

for(i in 1:n_growth_obs){

  # Equation 21: expected size at age a
  W_hat[i] <- xinf * (1 - exp(-gk * (age[i] - d0) - S_t[i] + S_t0)) +
    growth_ranef[growth_year[i]]

  S_t[i] <- (A * gk / (2 * pi)) * sin(2 * pi * (age[i] - ds))

  # Equation 22: variation in growth rate
  W[i] ~ dlnorm(log(W_hat[i]), sdlog = sigma_w)
}

# Equation 21: expected size at age a
S_t0 <- (A * gk / (2 * pi)) * sin(2 * pi * (d0 - ds))
```

```
for(y in 1:n_growth_years){

  growth_ranef[y] ~ dnorm(0, sd = sigma_y)

}

#######################
# Mark-recapture data #
#######################

# Equation 23: get number marked at first recapture event
S_mc_rc[1, 1:n_size] <- survival(alpha, beta, x[1:n_size],
                                 D_mc_rc[1], D_mc_rc[2])
total_marked_rc[1, 1:n_size] <- get_kernel(xinf, gk, sigma_G, A, ds,
                                           D_mc_rc[1], D_mc_rc[2],
                                           n_size, pi, x[1:n_size],
                                           lower[1:n_size],
                                           upper[1:n_size],
                                           S_mc_rc[1, 1:n_size]) %*%
  marked_rc[1, 1:n_size]

# Equation 24: project marked crab population with growth and mortality
for (t in 2:n_time_recap) { # time corresponds to marking events

  ## apply kernel from time of marking to time of recapture
  total_marked_rc[t, 1:n_size] <- get_kernel(xinf, gk, sigma_G, A, ds,
                                             D_mc_rc[t], D_mc_rc[t + 1],
                                             n_size, pi, x[1:n_size],
                                             lower[1:n_size],
                                             upper[1:n_size],
                                             S_mc_rc[t, 1:n_size]) %*%
    (total_marked_rc[t - 1, 1:n_size] + marked_rc[t, 1:n_size])

  S_mc_rc[t, 1:n_size] <- survival(alpha, beta, x[1:n_size],
                                   D_mc_rc[t], D_mc_rc[t + 1])

}

# Equation 25
## draw recaptured samples, catch_rc, from total marked samples, total_marked_rc
for (t in 1:n_time_recap) { # time corresponds to recapture events

  for (k in 1:n_size) {

    catch_rc[t, k] ~ dbinom(size = round(total_marked_rc[t, k]),
                            prob = p_mc_rc[t, k])

  }

  # Equation 26
  ## total capture probability with mark-recapture data
  p_mc_rc[t, 1:n_size] <- calc_prob(totalo_mc_rc[t + 1], n_size,
                                    hazard_mc_rc[t, 1:totalo_mc_rc[t + 1],
```

```
                                                          1:n_size])

    # size-dependent hazard rates with mark-recapture data
    hazard_mc_rc[t, 1:totalo_mc_rc[t + 1], 1:n_size] <- calc_hazard(
      totalo_mc_rc[t + 1], n_size, h_F_max, h_F_k, h_F_0, h_S_max, h_S_k,
      h_S_0, h_M_max, h_M_A, h_M_sigma,
      f_index_mc_rc[t + 1, 1:totalo_mc_rc[t + 1]],
      s_index_mc_rc[t + 1, 1:totalo_mc_rc[t + 1]],
      m_index_mc_rc[t + 1, 1:totalo_mc_rc[t + 1]],
      soak_days_mc_rc[t + 1, 1:totalo_mc_rc[t + 1]],
      x[1:n_size]
    )
  }


  #######################
  # Prior distributions #
  #######################

  ##
  # growth model
  ##

  # growth rate
  gk ~ dunif(0, 2)
  # amplitude of growth oscillations
  A ~ dunif(0, 4)
  # inflection point of growth oscillations
  ds ~ dunif(0, 1)
  # age organism has 0 size
  d0 ~ dunif(-10, 10)
  # process error sd
  sigma_w ~ dunif(0, 100)
  # year random effect sd
  sigma_y ~ dunif(0, 100)
  # asymptotic size
  xinf ~ dunif(70, 140)
  # growth error
  sigma_G ~ dunif(0.01, 4)

  ##
  # size selectivity parameters
  ##

  # minnow max. hazard rate
  h_M_max ~ dunif(0, 0.1)
  # minnow max. size of capture
  h_M_A ~ dunif(35, 60)
  # minnow sigma of gaussian size selectivity curve
  h_M_sigma ~ dunif(3, 10)
  # fukui max. hazard rate
  h_F_max ~ dunif(0, 0.1)
  # fukui k of logistic size selectivity curve
```

```
  h_F_k ~ dunif(0.1, 1.5)
  # fukui midpoint of logistic size selectivity curve
  h_F_0 ~ dunif(30, 100)
  # shrimp max. hazard rate
  h_S_max ~ dunif(0, 0.1)
  # shrimp k of logistic size selectivity curve
  h_S_k ~ dunif(0.1, 1.5)
  # shrimp midpoint of logistic size selectivity curve
  h_S_0 ~ dunif(30, 100)

  ##
  # IPM - natural mortality
  ##

  # size-independent natural mortality, shared across all years
  beta ~ dunif(0, 10000)
  # size-dependent natural mortality, shared across all years
  alpha ~ dunif(0, 10000)
  # size-dependent instantaneous prob of overwinter mortality, shared all years
  alpha_o ~ dunif(0, 50)
  # sd of instantaneous prob of overwinter mortality year-specific random effect
  sigma_o ~ dunif(0, 1000)

  ##
  # observation process
  ##

  # dirichlet multinomial (overdispersion in count data)
  ro_dir ~ dbeta(1, 1)
  n_p_dir <- (1 - ro_dir) / ro_dir

  ##
  # initial population density and annual recruitment
  ##

  # initial adult size (lognormal mean and sd)
  log_mu_A ~ dunif(3.25, 4.5)
  sigma_A ~ dunif(0.1, 1)

  # initial recruit size (mean and sd)
  mu_R ~ dunif(1, 25)
  sigma_R ~ dunif(0.01, 20)

  # abundance of recruits (lognormal mean and sd)
  mu_lambda ~ dunif(-50, 50)
  sigma_lambda ~ dunif(0, 10000)

  # abundance of adults in year 1
  lambda_A ~ dunif(1, 1000000)


})
```

Then declare the model data and constants, as well as generate initial values for the MCMC:

```r
# bundle up data and constants
constants <- list(
  # number of years
  n_year = dim(C)[3],
  # number of time periods in each year
  totalt = totalt,
  # number of trap obs in year y, time t
  totalo = totalo,
  # time periods to project all years to the same intra-annual end point
  additionalt = additionalt,
  # year indices where intra-annual end point > t of last observation
  year_short = which(apply(index, 2, max, na.rm = TRUE) <
                       max(index, na.rm = TRUE)),
  # number of years where intra-annual end point > t of last observation
  n_year_short = length(which(apply(index, 2, max, na.rm = TRUE) <
                                max(index, na.rm = TRUE))),
  # number of sizes in IPM mesh
  n_size = length(x),
  # upper bounds in IPM mesh
  upper = b[2:length(b)],
  # lower bounds in IPM mesh
  lower = b[1:length(x)],
  # binary indicator of fukui traps in time t, obs j, year i
  f_index = f_index,
  # binary indicator of minnow traps in time t, obs j, year i
  m_index = m_index,
  # binary indicator of shrimp traps in time t, obs j, year i
  s_index = s_index,
  # midpoints in IPM mesh
  x = x,
  # calendar date indices (fraction of year) in time t, year i
  D = D,
  # maximum calendar date index
  max_D = max(D, na.rm = TRUE),
  # number of soak days for each trap at time t, trap j, year i
  soak_days = soak_days,
  # data structure to introduce recruits into the model at t = 6
  recruit_intro = recruit_intro,
  # RC: total number of trap observations in mark-recapture dataset
  totalo_mc_rc = totalo_mc_rc,
  # RC: binary indicator of fukui traps in trap j of mark-recapture dataset
  f_index_mc_rc = f_index_mc_rc,
  # RC: binary indicator of shrimp traps in trap j of mark-recapture dataset
  s_index_mc_rc = s_index_mc_rc,
  # RC: binary indicator of minnow traps in trap j of mark-recapture dataset
  m_index_mc_rc = m_index_mc_rc,
  # RC: number of soak days in trap j of mark-recapture dataset
  soak_days_mc_rc = soak_days_mc_rc,
  # RC: calendar date indices (fraction of year) of mark-recapture dataset
  D_mc_rc = D_mc_rc,
  # number of RC recapture events
  n_time_recap = length(D_mc_rc) - 1,
```

```r
  pi = pi,
  ones = rep(1, length(x)),
  # growth data: number of crabs
  n_growth_obs = length(growth_data$CW),
  # growth data: year index
  growth_year = growth_data$year_index,
  # growth data: number of years
  n_growth_years = length(unique(growth_data$year_index))
)

data <- list(
  # total harvested within at time t, year y, size x
  C_T = C_T,
  # harvest count at time t, trap j, year y, size x
  C = C,
  # count of marked crabs at time t of size x in RC mark-recapture dataset
  marked_rc = marked_rc,
  # count of recaptured crabs at time t of size x in RC mark-recapture dataset
  catch_rc = catch_rc,
  # growth data: size captured
  W = growth_data$CW,
  # growth data: age
  age = growth_data$age
)

# create N_overwinter initial values
N_overwinter <- matrix(NA, nrow = dim(C)[3] - 1, ncol = length(x))
adult_mean <- c(log(75), log(80), log(82))
mean_recruit <- c(log(55), log(52), log(52))
lambda_A <- c(500, 400, 100)
lambda_R <- c(300, 75, 400)
adult_sd <- 0.08
recruit_sd <- 0.1
for (i in 1:(dim(C)[3] - 1)) {
  prob_r <- dlnorm(x, mean_recruit[i], recruit_sd) /
    sum(dlnorm(x, mean_recruit[i], recruit_sd))
  prob_a <- dlnorm(x, adult_mean[i], adult_sd) /
    sum(dlnorm(x, adult_mean[i], adult_sd))
  N_overwinter[i, ] <- round(prob_a * lambda_A[i] + prob_r * lambda_R[i])
}

# initial values
inits <- function() {
  list(
    h_M_max = runif(1, 0.0001, 0.0008), h_M_A = runif(1, 35, 60),
    h_M_sigma = runif(1, 5, 8), h_F_max = runif(1, 0.0001, 0.0008),
    h_F_k = runif(1, 0.1, 0.5), h_F_0 = runif(1, 30, 60),
    h_S_max = runif(1, 0.001, 0.005), h_S_k = runif(1, 0.1, 0.5),
    h_S_0 = runif(1, 30, 60), ro_dir = 0.01, alpha = 0.1,
    sigma_o = 0.5, eps_y = c(0, 0, 0), gk = 1,
    xinf = 85, A = 0.79, ds = 0.64, sigma_G = 2.5,
    sigma_R = 1, mu_R = 20, log_mu_A = 4, sigma_A = 0.2,
    lambda_A = 1800, lambda_R = c(1000, 100, 1000, 100), mu_lambda = log(500),
```

```
    sigma_lambda = 0.3, beta = 0.001,
    alpha_o = 0.0308, N_overwinter = N_overwinter,
    d0 = runif(1, -0.5, 0), sigma_w = runif(1, 0.01, 1),
    sigma_y = runif(1, 0.01, 1),
    growth_ranef = runif(length(unique(growth_data$year_index)), 0, 1)
  )
}
```

Run the MCMC in parallel, including assigning the relevant nimble functions to the parallel nodes:

```
########################
# run MCMC in parallel #
########################

cl <- makeCluster(4)

set.seed(10120)

clusterExport(cl, c("model_code", "inits", "data", "constants", "N_overwinter",
                    "growth_data"))

# Create a function with all the needed code
out <- clusterEvalQ(cl, {
  library(nimble)
  library(coda)
  library(expm)

  # define dirichlet multinomial mixture
  # pdf
  ddirchmulti <- nimbleFunction (
    run = function(x = double(1), alpha = double(1), size = double(0),
                   log = integer(0, default = 0)) {
      returnType(double(0))
      logProb <- lgamma(size + 1) - sum(lgamma(x + 1)) + lgamma(sum(alpha)) -
        sum(lgamma(alpha)) + sum(lgamma(alpha + x)) -
        lgamma(sum(alpha) + size)
      if (log) return(logProb)
      else return(exp(logProb))
    })
  # distribution number generator
  rdirchmulti <- nimbleFunction (
    run = function(n = integer(0), alpha = double(1), size = double(0)) {
      returnType(double(1))
      if (n != 1) print("rdirchmulti only allows n = 1; using n = 1.")
      p <- rdirch(1, alpha)
      return(rmulti(1, size = size, prob = p))
    })

  assign("ddirchmulti", ddirchmulti, .GlobalEnv)
  assign("rdirchmulti", rdirchmulti, .GlobalEnv)

  # size selective hazard rate of trap type minnow - bell-shaped curve
  size_sel_norm <- nimbleFunction (
    # input and output types
```

```
  run = function(pmax = double(0), xmax = double(0), sigma = double(0),
                 x = double(1))
  {
    returnType(double(1))

    vector <- pmax * exp(-(x - xmax) ^ 2 / (2 * sigma ^ 2))

    return(vector)
  }
)
assign("size_sel_norm", size_sel_norm, envir = .GlobalEnv)

# size selective hazard rate of trap type fukui and shrimp - logistic
size_sel_log <- nimbleFunction (
  #input and output types
  run = function(pmax = double(0), k = double(0), midpoint = double(0),
                 x = double(1))
  {
    returnType(double(1))

    vector <- pmax / (1 + exp(-k * (x - midpoint)))

    return(vector)
  }
)
assign("size_sel_log", size_sel_log, envir = .GlobalEnv)


# calculate trap hazard rate of obs j, based on trap type and soak days
calc_hazard <- nimbleFunction (
  #input and output types
  run = function(nobs = double(0), n_size = double(0), h_F_max = double(0),
                 h_F_k = double(0), h_F_0 = double(0),
                 h_S_max = double(0), h_S_k = double(0),
                 h_S_0 = double(0), h_M_max = double(0),
                 h_M_A = double(0), h_M_sigma = double(0),
                 obs_ref_f = double(1), obs_ref_s = double(1),
                 obs_ref_m = double(1), soak_days = double(1),
                 x = double(1))
  {
    returnType(double(2))

    # create empty array
    array <- array(init = FALSE, dim = c(nobs, n_size))
    # loop through observations
    for (j in 1:nobs) {
      # P(capture)
      array[j, 1:n_size] <- size_sel_log(pmax = h_F_max, k = h_F_k,
                                         midpoint = h_F_0, x = x) *
        obs_ref_f[j] * soak_days[j] +
        size_sel_log(pmax = h_S_max, k = h_S_k, midpoint = h_S_0, x = x) *
        obs_ref_s[j] * soak_days[j] +
        size_sel_norm(pmax = h_M_max, xmax = h_M_A,
```

```
                     sigma = h_M_sigma, x = x) *
        obs_ref_m[j] * soak_days[j]
    }

    return(array)
  }
)
assign("calc_hazard", calc_hazard, envir = .GlobalEnv)

# calculate conditional probability of capture
calc_cond_prob <- nimbleFunction (
  #input and output types
  run = function(nobs = double(0), n_size = double(0), hazard = double(2))
  {
    returnType(double(2))

    # create empty array
    array <- array(init = FALSE, dim = c(nobs, n_size))

    # loop through sizes
    for (k in 1:n_size) {
      #P(capture in trap j | captured at all)
      array[1:nobs, k] <- hazard[1:nobs, k] / sum(hazard[1:nobs, k])
    }
    return(array)
  }
)
assign("calc_cond_prob", calc_cond_prob, envir = .GlobalEnv)


# calculate total capture probability
calc_prob <- nimbleFunction (
  #input and output types
  run = function(nobs = double(0), n_size = double(0), hazard = double(2))
  {
    returnType(double(1))

    # create empty array
    p <- rep(NA, n_size)

    # loop through sizes
    for (k in 1:n_size) {
      # 1 - exp(-P(captured at all))
      p[k] <- 1 - exp(-sum(hazard[1:nobs, k]))
    }
    return(p)
  }
)
assign("calc_prob", calc_prob, envir = .GlobalEnv)

# function for seasonal growth kernel -- biweekly time step
get_kernel <- nimbleFunction (
  # input and output types
```

```
  run = function(xinf = double(0), k = double(0),
                 sigma_G = double(0), A = double(0),
                 ds = double(0), t1 = double(0), t2 = double(0),
                 n_size = double(0), pi = double(0), x = double(1),
                 lower = double(1), upper = double(1), S = double(1))
  {
    returnType(double(2))

    # create empty array
    array <- matrix(NA, ncol = n_size, nrow = n_size)

    if(!is.na(t2) & t2 == 0) {
      array <- diag(n_size)
    } else {
      # season adjusted params
      S_t <- (A * k / (2 * pi)) * sin(2 * pi * (t2 - ds))
      S_t0 <- (A * k / (2 * pi)) * sin(2 * pi * (t1 - ds))

      # p(y"|y)
      for (i in 1:n_size) {
        increment <- (xinf - x[i]) *
          (1 - exp(-k * (t2 - t1) - S_t + S_t0))
        mean <- x[i] + increment
        array[1:n_size, i] <- (pnorm(upper, mean, sd = sigma_G) -
                                 pnorm(lower, mean, sd = sigma_G))
      }

      # normalize and apply natural mortality
      for (i in 1:n_size) {
        array[, i] <- array[, i] / sum(array[, i]) * S
      }
    }
    return(array)
  }
)
assign("get_kernel", get_kernel, envir = .GlobalEnv)

# initial size distribution of adults - year 1
get_init_adult <- nimbleFunction (

  run = function(log_mu_A = double(0), sigma_A = double(0),
                 lower = double(1), upper = double(1),
                 lambda_A = double(0))
  {
    returnType(double(1))

    prop_adult <- plnorm(q = upper, meanlog = log_mu_A, sdlog = sigma_A) -
      plnorm(q = lower, meanlog = log_mu_A, sdlog = sigma_A)

    # get initial size-structured abundance of adults
    out <- prop_adult * lambda_A

    return(out)
```

```
  }
)
assign("get_init_adult", get_init_adult, envir = .GlobalEnv)

# annual size distributions of recruit
get_init_recruits <- nimbleFunction (

  run = function(mu_R = double(0), sigma_R = double(0),
                 lower = double(1), upper = double(1),
                 lambda_R = double(1), n_year = double(0),
                 n_size = double(0))
  {
    returnType(double(2))

    # create empty array
    out <- matrix(NA, ncol = n_size, nrow = n_year)

    # moment match from normal to gamma
    var <- sigma_R ^ 2
    shape <- mu_R ^ 2 / var
    rate <- mu_R / var
    prop_recruit <- pgamma(q = upper, shape = shape, rate = rate) -
      pgamma(q = lower, shape = shape, rate = rate)

    # get initial size-structured abundance of recruits
    for (y in 1:n_year) {
      out[y, ] <- prop_recruit[1:n_size] * lambda_R[y]
    }

    return(out)
  }
)
assign("get_init_recruits", get_init_recruits, envir = .GlobalEnv)

# natural (non-winter) survival
survival <- nimbleFunction (

  run = function(alpha = double(0), beta = double(0), x = double(1),
                 t1 = double(0), t2 = double(0))
  {
    returnType(double(1))

    # number of biweeks
    deltat <- round((t2 - t1) * (52.1429 / 2))

    # get survival rate
    out <- exp(-deltat * (beta + alpha / x ^ 2))

    return(out)
  }
)
assign("survival", survival, envir = .GlobalEnv)
```

```r
# density- and size-dependent overwinter survival
overwinter_survival <- nimbleFunction (

  run = function(alpha_o = double(0), N_sum = double(0),
                 x = double(1), eps_y = double(0))
  {
    returnType(double(1))

    # get probability of survival
    out <- exp(-(alpha_o * N_sum / x ^ 2 + eps_y))

    return(out)
  }
)
assign("overwinter_survival", overwinter_survival, envir = .GlobalEnv)


# build model
myModel <- nimbleModel(code = model_code,
                       data = data,
                       constants = constants,
                       inits = inits())


# build the MCMC
mcmcConf_myModel <- configureMCMC(
  myModel,
  monitors = c("h_M_max", "h_M_A", "h_M_sigma", "h_F_max",
               "h_F_k", "h_F_0", "h_S_max", "h_S_k",
               "h_S_0", "gk",
               "xinf", "A", "ds", "sigma_G",
               "sigma_R", "mu_R", "log_mu_A",
               "sigma_A", "mu_lambda", "sigma_lambda",
               "lambda_R", "lambda_A", "beta", "alpha_o",
               "alpha", "eps_y", "sigma_o", "N_overwinter",
               "wgrowth_N_sum", "ro_dir", "C_T",
               "", "sigma_w", "sigma_y", "growth_ranef"),
  useConjugacy = FALSE, enableWAIC = TRUE)

# build MCMC
myMCMC <- buildMCMC(mcmcConf_myModel)

# compile the model and MCMC
CmyModel <- compileNimble(myModel)

# compile the MCMC
cmodel_mcmc <- compileNimble(myMCMC, project = myModel)

# run MCMC
cmodel_mcmc$run(100000, thin = 10,
                reset = FALSE)

samples <- as.mcmc(as.matrix(cmodel_mcmc$mvSamples))
```

```r
  return(samples)
})
```

Finally, discard burn-in, save the samples, and stop the cluster:

```r
# discard burnin
lower <- 2000
upper <- 10001
sequence <- seq(lower, upper, 1)
out_sub <- list(out[[1]][sequence, ], out[[2]][sequence, ],
                out[[3]][sequence, ], out[[4]][sequence, ])

# save samples
saveRDS(out_sub, "data/posterior_samples/savedsamples_IPM.rds")

stopCluster(cl)
```
