# Supplementary material for: An integrated integral projection model (IPM2 ) to disentangle size‐structured harvest and natural mortality
Source: J Anim Ecol. 2025 Nov 11;95(1):157–74. doi: 10.1111/1365-2656.70176 (PMC12775557; doi:10.1111/1365-2656.70176)
Supplement: Supplementary file 4 — Appendix 4. Generic model code. [file JANE-95-157-s006.pdf]

Appendix 4: Generic model code

Here we provide generic model code that closely follows the model description described in the main text, without the extra data structures to account for data heterogeneity (as found in Appendix 3).

Generic model code

```
model_code <- NimbleCode({

  #################
  #################
  # Process model #
  #################
  #################

  ##############################
  # Integral projection model #
  ##############################

  # intra-annual change
  for (t in 1:(Tmax-1)) {
    for (y in 1:n_year) {

      # Equation 1
      ## project
      N[t + 1, y, 1:n_size] <- kernel[1:n_size, 1:n_size, t] %*%
        (N[t, y, 1:n_size] - C_T[t, y, 1:n_size]) +
        recruit_intro[t, y] * R[y, 1:n_size] # introduce recruits, t = 6
    }
    # Equation 2
    ## kernel
    kernel[1:n_size, 1:n_size, t] <- seasonal_growth(
      xinf, gk, sigma_G, A, ds, D[t, 1:2], n_size, pi, x[1:n_size],
      lower[1:n_size], upper[1:n_size]) * S[t, 1:n_size]

    # Equation 7
    ## natural survival
    S[t, 1:n_size] <- survival(alpha, beta, D[t, 1:2], x[1:n_size])
  }

  ## inter-annual change
  for (y in 1:(n_year - 1)) {
    for (k in 1:n_size) {

      # Equation 8, 11
```

```
    ## overwinter survival and seasonal growth
    ## (use stochastic N node)
    N_stoch[y, k] ~ dbinom(size = seasonal_growth(xinf, gk, sigma_G, A, ds,
                                                  D[Tmax, 1:2], k, pi, y[k],
                                                  lower[k], upper[k]) %*%
                              (N[Tmax, y, k] - C_T[Tmax, y, k]),
                            prob = S_o[y, k])

  }

  # fill deterministic N node with stochastic N node
  N[1, y + 1, 1:n_size] <- N_stoch[y, 1:n_size]

  # Equation 9
  ## size- and density-dependent overwinter survival
  S_o[y, 1:n_size] <- overwinter_survival(alpha_o[y],
                                          sum(N[Tmax, y, 1:n_size]),
                                          x[1:n_size], eps_y[y])

  # Equation 10
  ## year-specific overwinter mortality random effect
  eps_y[y] ~ dnorm(0, sd = sigma_o)
}

#####################################################
# Initial population density and annual recruitment #
#####################################################

# Equation 12
## get initial size-structured abundance of adults - year 1
N[1, 1, 1:n_size] <- get_init_adult(mu_A, sigma_A, lower[1:n_size],
                                    upper[1:n_size], lambda_A)

# Equation 13
## annual abundance of recruits
for (m in 1:n_year) {
  lambda_R[m] ~ dlnorm(mu_lambda, sdlog = sigma_lambda)
}

# Equation 14
## annual size distribution of recruits
R[1:n_year, 1:n_size] <- get_init_recruits(mu_R, sigma_R, lower[1:n_size],
                                           upper[1:n_size],
                                           lambda_R[1:n_year], n_year,
                                           n_size)

#####################
#####################
# Observation model #
#####################
#####################

for (y in 1:n_year) {
```

```
  for (t in 1:Tmax) {
    for (k in 1:n_size) {

      # Equation 15
      ## binomial distribution with total crabs removed, n_R
      C_T[t, y, k] ~ dbinom(size = round(N[t, y, k]), prob = p[t, y, k])

      # Equation 16
      ## dirichlet-multinomial mixture, conditional probability of capture
      alpha_D[t, 1:totalo, y, k] <- p_C[t, 1:totalo, y, k] * n_p_dir
      C[t, 1:totalo, y, k] ~ ddirchmulti(alpha = alpha_D[t, 1:totalo, y, k],
                                         size = C_T[t, y, k])
    }

    # Equations 17 - 19
    ## calculate hazard rate
    hazard[t, 1:totalo, y, 1:n_size] <- calc_hazard(
      totalo, n_size, h_F_max, h_F_k, h_F_0, h_S_max, h_S_k, h_S_0, h_M_max,
      h_M_A, h_M_sigma, f_index[t, 1:totalo, y], s_index[t, 1:totalo, y],
      m_index[t, 1:totalo, y], soak_days[t, 1:totalo, y], x[1:n_size]
      )

    # Equation 20
    ## total capture probability
    p[t, y, 1:n_size] <- calc_prob(totalo, n_size,
                                   hazard[t, 1:totalo, y, 1:n_size])

    # mean conditional probability of capture
    p_C[t, 1:totalo, y, 1:n_size] <- calc_cond_prob(totalo, n_size,
                                                    hazard[t, 1:totalo,
                                                           y, 1:n_size])

  }
}


###############################
###############################
# Integrated population model #
###############################
###############################

################
# Growth model #
################

for(i in 1:n_growth_obs){

  # Equation 21: expected size at age a
  W_hat[i] <- xinf * (1 - exp(-gk * (age[i] - d0) - S_t[i] + S_t0)) +
    growth_ranef[growth_year[i]]

  S_t[i] <- (A * gk / (2 * pi)) * sin(2 * pi * (age[i] - ds))
```

```
  # Equation 22: variation in growth rate
  W[i] ~ dlnorm(log(W_hat[i]), sdlog = sigma_w)
}

# Equation 21: expected size at age a
S_t0 <- (A * gk / (2 * pi)) * sin(2 * pi * (d0 - ds))

for(y in 1:n_growth_years){

  growth_ranef[y] ~ dnorm(0, sd = sigma_y)

}

#######################
# Mark-recapture data #
#######################

# Equation 23: get number marked at first recapture event
S_mc_rc[1, 1:n_size] <- survival(alpha, beta, x[1:n_size],
                                 D_mc_rc[1], D_mc_rc[2])
total_marked_rc[1, 1:n_size] <- get_kernel(xinf, gk, sigma_G, A, ds,
                                           D_mc_rc[1], D_mc_rc[2],
                                           n_size, pi, x[1:n_size],
                                           lower[1:n_size],
                                           upper[1:n_size],
                                           S_mc_rc[1, 1:n_size]) %*%
  marked_rc[1, 1:n_size]

# Equation 24: project marked crab population with growth and mortality
for (t in 2:n_time_recap) { # time corresponds to marking events

  ## apply kernel from time of marking to time of recapture
  total_marked_rc[t, 1:n_size] <- get_kernel(xinf, gk, sigma_G, A, ds,
                                             D_mc_rc[t], D_mc_rc[t + 1],
                                             n_size, pi, x[1:n_size],
                                             lower[1:n_size],
                                             upper[1:n_size],
                                             S_mc_rc[t, 1:n_size]) %*%
    (total_marked_rc[t - 1, 1:n_size] + marked_rc[t, 1:n_size])

  S_mc_rc[t, 1:n_size] <- survival(alpha, beta, x[1:n_size],
                                   D_mc_rc[t], D_mc_rc[t + 1])

}

# Equation 25
## draw recaptured samples, catch_rc, from total marked samples, total_marked_rc
for (t in 1:n_time_recap) { # time corresponds to recapture events

  for (k in 1:n_size) {

    catch_rc[t, k] ~ dbinom(size = round(total_marked_rc[t, k]),
                           prob = p_mc_rc[t, k])
```

```
  }

  # Equation 26
  ## total capture probability with mark-recapture data
  p_mc_rc[t, 1:n_size] <- calc_prob(totalo_mc_rc[t + 1], n_size,
                                    hazard_mc_rc[t, 1:totalo_mc_rc[t + 1],
                                                 1:n_size])

  # size-dependent hazard rates with mark-recapture data
  hazard_mc_rc[t, 1:totalo_mc_rc[t + 1], 1:n_size] <- calc_hazard(
    totalo_mc_rc[t + 1], n_size, h_F_max, h_F_k, h_F_0, h_S_max, h_S_k,
    h_S_0, h_M_max, h_M_A, h_M_sigma,
    f_index_mc_rc[t + 1, 1:totalo_mc_rc[t + 1]],
    s_index_mc_rc[t + 1, 1:totalo_mc_rc[t + 1]],
    m_index_mc_rc[t + 1, 1:totalo_mc_rc[t + 1]],
    soak_days_mc_rc[t + 1, 1:totalo_mc_rc[t + 1]],
    x[1:n_size]
  )
}


#######################
# Prior distributions #
#######################

##
# growth model
##

# growth rate
gk ~ dunif(0, 2)
# amplitude of growth oscillations
A ~ dunif(0, 4)
# inflection point of growth oscillations
ds ~ dunif(0, 1)
# age organism has 0 size
d0 ~ dunif(-10, 10)
# process error sd
sigma_w ~ dunif(0, 100)
# year random effect sd
sigma_y ~ dunif(0, 100)
# asymptotic size
xinf ~ dunif(70, 140)
# growth error
sigma_G ~ dunif(0.01, 4)

##
# size selectivity parameters
##

# minnow max. hazard rate
h_M_max ~ dunif(0, 0.1)
# minnow max. size of capture
```

```
h_M_A ~ dunif(35, 60)
# minnow sigma of gaussian size selectivity curve
h_M_sigma ~ dunif(3, 10)
# fukui max. hazard rate
h_F_max ~ dunif(0, 0.1)
# fukui k of logistic size selectivity curve
h_F_k ~ dunif(0.1, 1.5)
# fukui midpoint of logistic size selectivity curve
h_F_0 ~ dunif(30, 100)
# shrimp max. hazard rate
h_S_max ~ dunif(0, 0.1)
# shrimp k of logistic size selectivity curve
h_S_k ~ dunif(0.1, 1.5)
# shrimp midpoint of logistic size selectivity curve
h_S_0 ~ dunif(30, 100)

##
# IPM - natural mortality
##

# size-independent natural mortality, shared across all years
beta ~ dunif(0, 10000)
# size-dependent natural mortality, shared across all years
alpha ~ dunif(0, 10000)
# size-dependent instantaneous prob of overwinter mortality, shared all years
alpha_o ~ dunif(0, 50)
# sd of instantaneous prob of overwinter mortality year-specific random effect
sigma_o ~ dunif(0, 1000)

##
# observation process
##

# dirichlet multinomial (overdispersion in count data)
ro_dir ~ dbeta(1, 1)
n_p_dir <- (1 - ro_dir) / ro_dir

##
# initial population density and annual recruitment
##

# initial adult size (lognormal mean and sd)
log_mu_A ~ dunif(3.25, 4.5)
sigma_A ~ dunif(0.1, 1)

# initial recruit size (mean and sd)
mu_R ~ dunif(1, 25)
sigma_R ~ dunif(0.01, 20)

# abundance of recruits (lognormal mean and sd)
mu_lambda ~ dunif(-50, 50)
sigma_lambda ~ dunif(0, 10000)
```

```
  # abundance of adults in year 1
  lambda_A ~ dunif(1, 1000000)


})
```

Nimble functions

Process model functions

Seasonal growth

This function corresponds to equations 5-6 in main text.

```
# function for seasonal growth kernel
seasonal_growth <- nimbleFunction (
  run = function(xinf = double(0), k = double(0),
                 sigma_G = double(0), A = double(0),
                 ds = double(0), D = double(1),
                 n_size = double(0), pi = double(0), x = double(1),
                 lower = double(1), upper = double(1))
  {
    returnType(double(2))

    # create empty array
    array <- matrix(NA, ncol = n_size, nrow = n_size)

    # season adjusted params
    S_t <- (A * k / (2 * pi)) * sin(2 * pi * (D[2] - ds))
    S_t0 <- (A * k / (2 * pi)) * sin(2 * pi * (D[1] - ds))

    # p(y"|y)
    for (i in 1:n_size) {
      increment <- (xinf - x[i]) *
        (1 - exp(-k * (D[2] - D[1]) - S_t + S_t0))
      mean <- x[i] + increment
      array[1:n_size, i] <- (pnorm(upper, mean, sd = sigma_G) -
                               pnorm(lower, mean, sd = sigma_G))
    }

    # normalize
    for (i in 1:n_size) {
      array[, i] <- array[, i] / sum(array[, i])
    }
  return(array)
}
)
```

Natural survival

This function corresponds to equation 7 in main text.

```
# natural (non-winter) survival
survival <- nimbleFunction (

  run = function(alpha = double(0), beta = double(0), D = double(1),
                 x = double(1))
  {
    returnType(double(1))

    # get survival rate
    out <- exp(-(D[2] - D[1]) * (beta + alpha / x ^ 2))

    return(out)
  }
)
```

Density-dependent overwinter survival

This function corresponds to equation 9 in main text.

```
# density- and size-dependent overwinter survival
overwinter_survival <- nimbleFunction (

  run = function(alpha_o = double(0), N = double(1),
                 x = double(1), eps_y = double(0))
  {
    returnType(double(1))

    # get probability of survival
    out <- exp(-(alpha_o * sum(N) / x ^ 2 + eps_y))

    return(out)
  }
)
```

Initial population density and annual recruitment

This function corresponds to equation 12 in main text.

```
# initial size distribution of adults - year 1
get_init_adult <- nimbleFunction (

  run = function(mu_A = double(0), sigma_A = double(0),
                 lower = double(1), upper = double(1),
                 lambda_A = double(0))
  {
    returnType(double(1))

    # moment match from normal to gamma
    var <- sigma_A ^ 2
    shape <- mu_A ^ 2 / var
    rate <- mu_A / var
    prop_adult <- pgamma(q = upper, shape = shape, rate = rate) -
```

```
      pgamma(q = lower, shape = shape, rate = rate)

    # get initial size-structured abundance of adults
    out <- prop_adult * lambda_A

    return(out)
  }
)
```

Size-structured recruitment

This function corresponds to equation 14 in main text.

```
# annual size distributions of recruit
get_init_recruits <- nimbleFunction (

  run = function(mu_R = double(0), sigma_R = double(0),
                 lower = double(1), upper = double(1),
                 lambda_R = double(1), n_year = double(0),
                 n_size = double(0))
  {
    returnType(double(2))

    # create empty array
    out <- matrix(NA, ncol = n_size, nrow = n_year)

    var <- sigma_R ^ 2
    shape <- mu_R ^ 2 / var
    rate <- mu_R / var
    prop_recruit <- pgamma(q = upper, shape = shape, rate = rate) -
      pgamma(q = lower, shape = shape, rate = rate)

    # get initial size-structured abundance of recruits
    for (i in 1:n_year) {
      out[i, ] <- prop_recruit[1:n_size] * lambda_R[i]
    }

    return(out)
  }
)
```

Observation model functions

Conditional multinomial observation model

This function corresponds to equations 17-19 in main text.

```
# calculate trap hazard rate of obs j, based on trap type and soak days
calc_hazard <- nimbleFunction (

  run = function(nobs = double(0), n_size = double(0), h_F_max = double(0),
                 h_F_k = double(0), h_F_0 = double(0), h_S_max = double(0),
```

```
                 h_S_k = double(0), h_S_0 = double(0), h_M_max = double(0),
                 h_M_A = double(0), h_M_sigma = double(0), f_index = double(1),
                 s_index = double(1), m_index = double(1),
                 soak_days = double(1), x = double(1))
  {
    returnType(double(2))

    # create empty array
    array <- array(init = FALSE, dim = c(nobs, n_size))

    # loop through observations
    for (j in 1:nobs) {
      # P(capture)
      array[j, 1:n_size] <- size_sel_log(pmax = h_F_max, k = h_F_k,
                                          midpoint = h_F_0, x = x) *
        f_index[j] * soak_days[j] +
        size_sel_log(pmax = h_S_max, k = h_S_k, midpoint = h_S_0, x = x) *
        s_index[j] * soak_days[j] +
        size_sel_norm(pmax = h_M_max, xmax = h_M_A, sigma = h_M_sigma, x = x) *
        m_index[j] * soak_days[j]
    }
      return(array)
  }
)
```

These functions correspond to equation 20 in main text

```
# calculate conditional probability of capture
calc_cond_prob <- nimbleFunction (
  run = function(nobs = double(0), n_size = double(0), hazard = double(2))
  {
    returnType(double(2))

    # create empty array
    array <- array(init = FALSE, dim = c(nobs, n_size))
    # loop through sizes
    for (k in 1:n_size) {
      #P(capture in trap j | captured at all)
      array[1:nobs, k] <- hazard[1:nobs, k] / sum(hazard[1:nobs, k])
    }
    return(array)
  }
)

# calculate total capture probability
calc_prob <- nimbleFunction (

  run = function(nobs = double(0), n_size = double(0), hazard = double(2))
  {
    returnType(double(1))

    # create empty array
    p <- rep(NA, n_size)
```

```
    # loop through sizes
    for (k in 1:n_size) {
      # 1 - exp(-P(captured at all))
      p[k] <- 1 - exp(-sum(hazard[1:nobs, k]))
    }
    return(p)
  }
)
```

Size-selective hazard rates

These functions correspond to equations 17-19 in main text.

```
# bell-shaped size selective hazard rate
size_sel_norm <- nimbleFunction (
  # input and output types
  run = function(pmax = double(0), xmax = double(0), sigma = double(0),
                 x = double(1))
  {
    returnType(double(1))
      vector <- pmax * exp(-(x - xmax) ^ 2 / (2 * sigma ^ 2))

    return(vector)
  }
)

# logistic size selective hazard rate
size_sel_log <- nimbleFunction (
  run = function(pmax = double(0), k = double(0), midpoint = double(0),
                 x = double(1))
  {
    returnType(double(1))

    vector <- pmax / (1 + exp(-k * (x - midpoint)))

    return(vector)
  }
)
```

Dirichlet-multinomial mixture

These functions correspond to equation 16 in main text.

```
# define dirichlet multinomial mixture
# pdf
ddirchmulti <- nimbleFunction (
  run = function(x = double(1), alpha = double(1), size = double(0),
                 log = integer(0, default = 0)) {
    returnType(double(0))
    logProb <- lgamma(size + 1) - sum(lgamma(x + 1)) + lgamma(sum(alpha)) -
      sum(lgamma(alpha)) + sum(lgamma(alpha + x)) -
```

```
      lgamma(sum(alpha) + size)
    if (log) return(logProb)
    else return(exp(logProb))
  })
# random number generator
rdirchmulti <- nimbleFunction (
  run = function(n = integer(0), alpha = double(1), size = double(0)) {
    returnType(double(1))
    if (n != 1) print("rdirchmulti only allows n = 1; using n = 1.")
    p <- rdirch(1, alpha)
    return(rmulti(1, size = size, prob = p))
  })
```
