# Supplementary material for: An integrated integral projection model (IPM2 ) to disentangle size‐structured harvest and natural mortality
Source: J Anim Ecol. 2025 Nov 11;95(1):157–74. doi: 10.1111/1365-2656.70176 (PMC12775557; doi:10.1111/1365-2656.70176)

Appendix 5: Posterior summaries and trace plots

Appendix 5

Table A5: Posterior summaries, including the mean, standard deviation, 95% credibility interval (highest density interval), $\hat{R}$, and effective sample size. Descriptions of parameters can be found in Table 1. More information about parameter ρ can be found in Appendix 2.1.

\

parameter	mean	sd	95 CI	Rhat	ESS
μ^A	3.798	0.045	(3.707, 3.883)	1.01	484
σ^A	0.2156	0.0191	(0.1789, 0.253)	1.01	676
μ^R	10.74	2.68	(5.19, 15.79)	1.03	462
σ_R	5.023	0.83	(3.515, 6.57)	1.00	1868
x_∞	80.5	0.91	(78.75, 82.31)	1.02	287
k	1.209	0.075	(1.07, 1.358)	1.02	316
A	1.538	0.162	(1.227, 1.837)	1.01	362
d_s	0.2437	0.0197	(0.2083, 0.2841)	1.01	512
σ_G	2.818	0.218	(2.38, 3.234)	1.03	214
d_0	0.1962	0.0289	(0.1383, 0.2524)	1.00	1562
σ_w	0.1091	0.0123	(0.0866, 0.1341)	1.00	10562
σ_u	4.552	1.807	(1.416, 8.345)	1.00	6363
β	0.00178	0.001727	(0, 0.005219)	1.00	1832
α	9.498	9.424	(0, 28.528)	1.01	860
α_o	3.037	0.525	(2.045, 4.08)	1.01	486
ϵ_1	0.01916	0.08396	(-0.15659, 0.2066)	1.00	2712

(continued)

parameter	mean	sd	95 CI	Rhat	ESS
ϵ_2	0.00137	0.040402	(-0.063462, 0.084062)	1.00	7129
ϵ_3	-0.0774	0.11252	(-0.33264, 0.08968)	1.00	3555
σ_o	0.166	0.2532	(0.001, 0.5377)	1.05	1884
h_F^{max}	0.0001784	1.5e-05	(0.0001498, 0.0002084)	1.00	3829
h_M^{max}	0.0003912	6.17e-05	(0.0002752, 0.0005132)	1.00	1903
h_S^{max}	0.003937	4e-04	(0.003186, 0.004752)	1.00	1888
h_F^k	0.4977	0.1878	(0.2244, 0.8818)	1.00	4791
h_S^k	0.3437	0.0543	(0.245, 0.4523)	1.00	5633
h_F^0	35.34	1.98	(31.65, 39.26)	1.00	1498
h_S^0	46.41	1.46	(43.55, 49.21)	1.00	1779
h_M^A	45.12	0.67	(43.82, 46.45)	1.00	1508
h_M^σ	6.449	0.376	(5.723, 7.188)	1.00	16250
ρ	0.03818	0.00737	(0.02397, 0.05268)	1.00	31822
λ^A	441.3	44	(360, 529.1)	1.00	2019
λ_1^R	697.6	237.8	(322.1, 1172.1)	1.02	1084
λ_2^R	50.32	13.04	(27.68, 75.8)	1.00	1712
λ_3^R	1394	344	(842, 2086)	1.01	852
λ_4^R	61.35	24.93	(21.47, 109.49)	1.01	2101
μ^λ	5.514	2.738	(0.706, 9.952)	1.00	8884
μ^σ	3.651	4.378	(0.765, 9.353)	1.02	3787

Figure A5: Trace plots of posterior samples. Colors refer to separate chains. Descriptions of parameters can be found in Table 1.

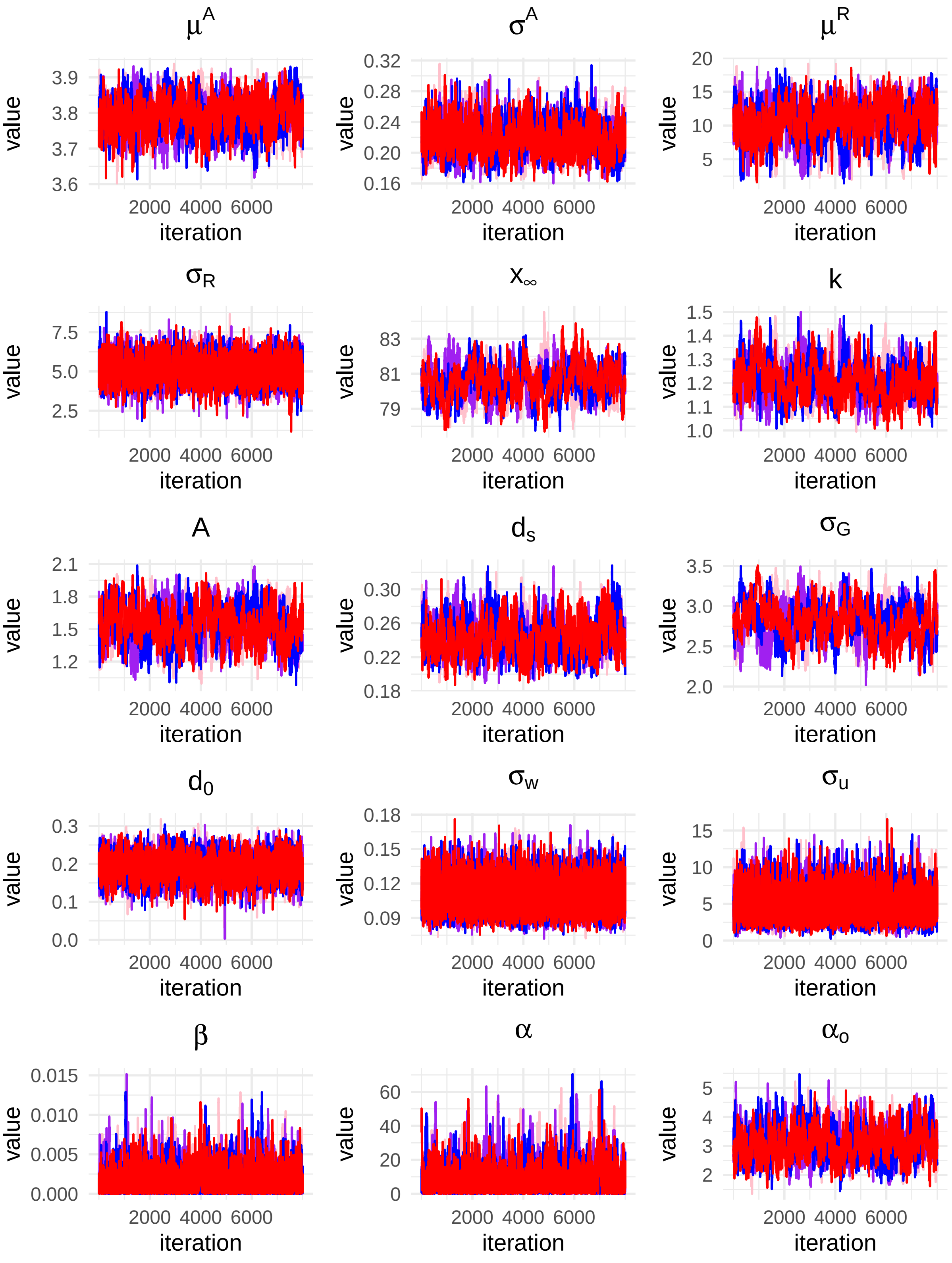

Figure A5 continued.

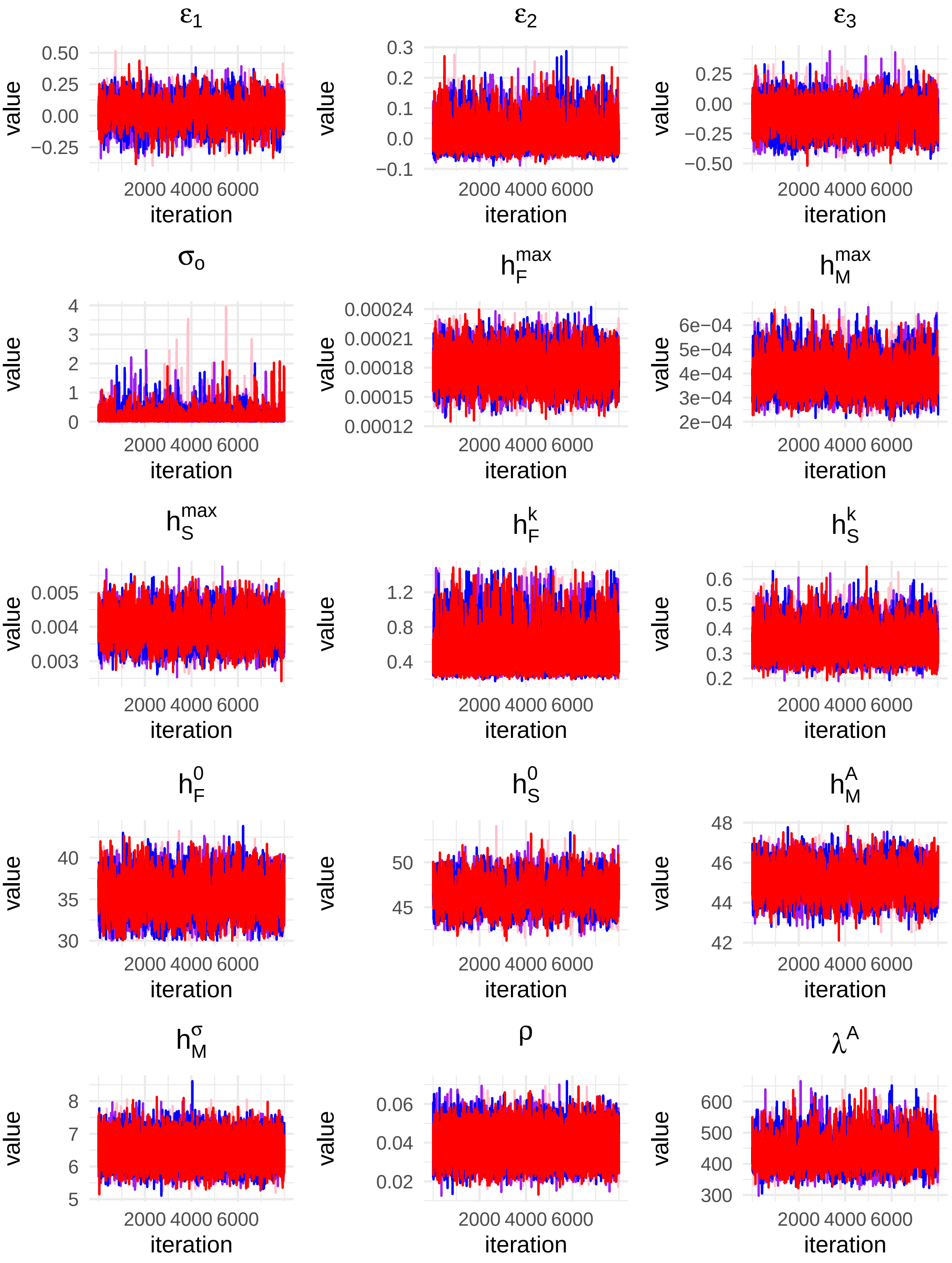

Figure A5 continued.

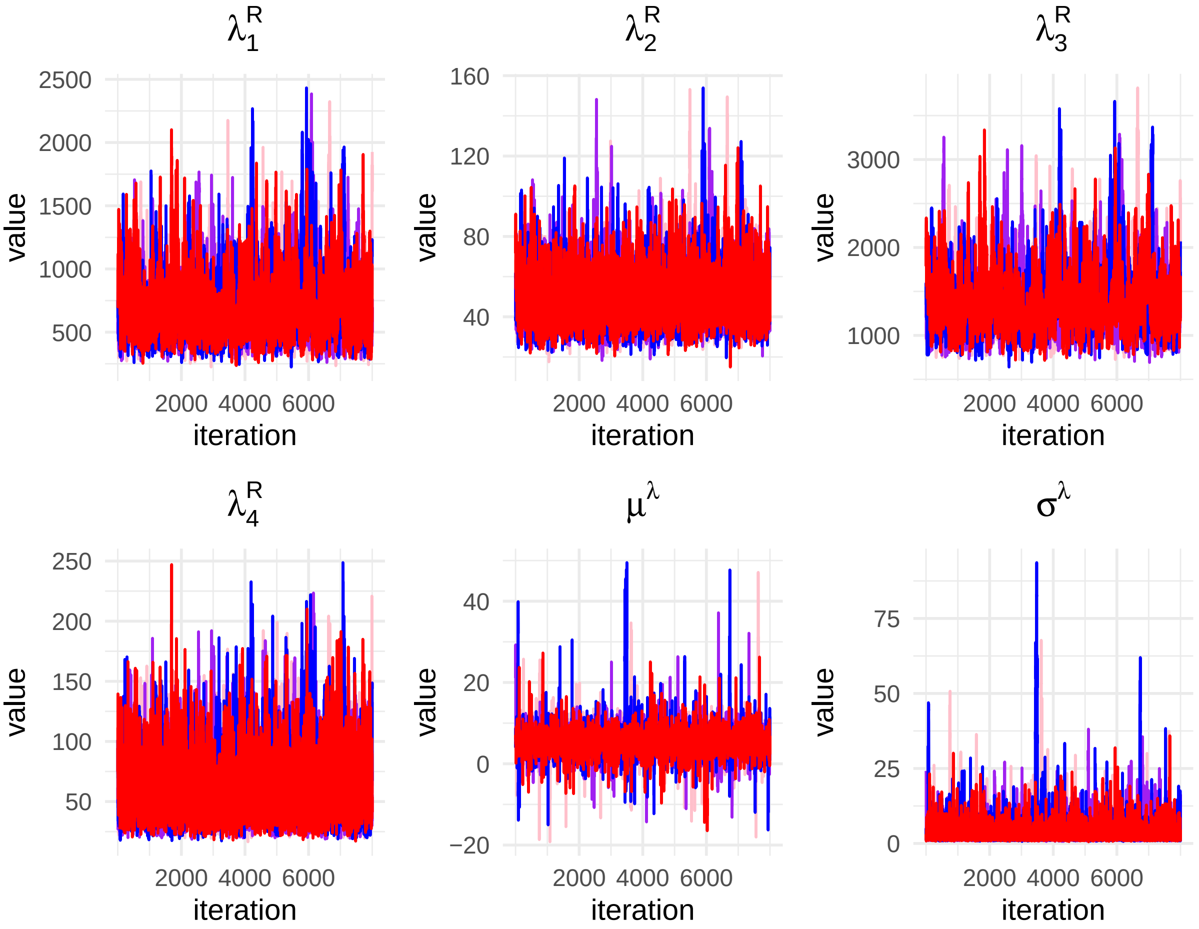

Supplement: Supplementary file 5 — Appendix 5. Posterior summaries and trace plots. [file JANE-95-157-s002.pdf]
