# Supplementary material for: An integrated integral projection model (IPM2 ) to disentangle size‐structured harvest and natural mortality
Source: J Anim Ecol. 2025 Nov 11;95(1):157–74. doi: 10.1111/1365-2656.70176 (PMC12775557; doi:10.1111/1365-2656.70176)
Supplement: Supplementary file 6 — Appendix 6. Robustness Assessments. [file JANE-95-157-s004.pdf]

Appendix 6: Robustness Assessments

Appendix 6

To assess model performance and robustness, we conducted both a model selection and model checking procedure. In Appendix 6.1, we describe the model selection procedure used to evaluate multiple functional forms for describing the overwinter mortality process using Watanabe–Akaike information criterion (WAIC). In Appendix 6.2, we describe the model checking procedure and calculation of posterior predictive p-values. In Appendix 6.3, we evaluate the model fitted with a different mark-recapture dataset (D3) from Seadrift Lagoon in California, USA to demonstrate how posterior predictive checks can be useful in assessing the feasibility of integrating disparate datasets in the integrated population model.

Appendix 6.1: Model selection

The inter-annual population transitions (i.e., transition from year y to year $y+1$) are largely described by density-dependent overwinter mortality. Since density dependence only enters the model during this process and is therefore likely influential for forecasting the stable size distribution, we compared multiple functional forms for size- and density-dependent overwinter mortality (Equation 9 in main text).

We fit four separate models that are identical, apart from the functional form of overwinter survival (Equation 9). These models varied by whether the relationship between size- and density-dependence is additive or interactive, as well as how rapidly mortality decreases with size.

Model 1: interactive density- and size-dependence, steeper mortality decrease with size

$$S_o(x, N^T_{t_{\max},y}) = \exp(-(\frac{\alpha^o \times N^T_{t_{\max},y}}{x^2}) + \epsilon_y)$$

Model 2: interactive density- and size-dependence, less steep mortality decrease with size

$$S_o(x, N^T_{t_{\max},y}) = \exp(-(\frac{\alpha^o \times N^T_{t_{\max},y}}{x}) + \epsilon_y)$$

Model 3: additive density- and size-dependence, steeper mortality decrease with size

$$S_o(x, N^T_{t_{\max},y}) = \exp(-(\frac{\alpha^o}{N^T_{t_{\max},y}} + \frac{\psi}{x^2} + \epsilon_y))$$

Model 4: additive density- and size-dependence, less steep mortality decrease with size

$$S_o(x, N^T_{t_{\max},i}) = \exp(-(\frac{\alpha^o}{N^T_{t_{\max},y}} + \frac{\psi}{x} + \epsilon_y))$$

WAIC was calculated using every 10th sample from the combined posterior samples from four chains (3200 total samples). Model 1, with a size- and density-dependent overwinter mortality interaction and a steeper mortality decrease with size had the lowest WAIC and was used in subsequent analyses.

Model	WAIC	lppd	pWAIC	ΔWAIC
Model 1	6776.36	-3339.95	48.17	0.00
Model 2	6783.83	-3341.68	50.23	7.47
Model 3	6807.36	-3472.75	66.43	31.00
Model 4	7078.36	-3472.75	66.43	302.00

Appendix 6.2

Posterior predictive checks (PPCs) were used to determine if the model is an adequate representation of the observed data. The PPCs are based on the intuition that data simulated under a fitted model should be similar to the observed data the model was fitted to (Conn et al. 2018). Calculating PPCs involves 1) cyclically drawing parameter values from the posterior distribution ($\boldsymbol{\theta}_i \sim [\boldsymbol{\theta}|\mathbf{D}]$), 2) generating a replicate data set for each i, $\mathbf{D}_i^{\text{rep}} \sim [\mathbf{D}|\boldsymbol{\theta}_i]$, 3) calculating a discrepancy test statistic, $T(\mathbf{D}, \boldsymbol{\theta})$, for $\mathbf{D}$ and $\mathbf{D}^{\text{rep}}$, and 4) comparing the distributions of test statistics of the observed data, $T(\mathbf{D}_i, \boldsymbol{\theta}_i)$, and replicated data, $T(\mathbf{D}_i^{\text{rep}}, \boldsymbol{\theta}_i)$. Bayesian p-values can be calculated based on these two distributions of test-statistics.

We used two discrepancy functions, $T(\mathbf{D}, \boldsymbol{\theta})$, to test goodness of fit. Note that $\boldsymbol{\theta}$ includes both top-level parameters in the model (i.e., x_∞, α, etc.), as well as latent states/random effects in the model (i.e., σ_y, λ_y^R, etc.).

The first discrepancy function is an omnibus discrepancy function, deviance, which tests global lack of fit and was used by King et al. 2009 (King et al. 2009). Here, $\mathbf{D}$ refers to the the count of removed crabs, $C_{t,j,y}(x_i)$ in the multi-year time-series data set (D1), the total number of removed crabs, $C_{t,i}^T(x_i)$ in the multi-year time-series data set (D1), the number of recaptured marked crabs, $r_t^{\text{mc}}(x_i)$ in the mark-recapture data set (D3), and the size of crabs in the size-at-age dataset (D2):

$$T(\mathbf{D}, \boldsymbol{\theta}) = -2\text{log}(\mathbf{D}|\boldsymbol{\theta})$$

The second discrepancy function is a targeted discrepancy function that calculates the proportion of zeros and checks zero inflation in the count data (Conn et al. 2018). We include this targeted measure since the count data in the multi-year time series data set (D1) is overdispersed due to spatial aggregation behavior of the crabs. Additionally, while posterior predictive P values are known to be conservative (Conn et al. 2018), previous work suggests that discrepancy functions that are solely a function of simulated and observed data may be less conservative than those that depend on model parameters, $\boldsymbol{\theta}$, like most omnibus discrepancy functions (Lunn et al. 2013).

Here, $\mathbf{D}$ refers to the the count of removed crabs, $C(x)_{t,j,y}$:

$$T(\mathbf{D}) = \sum_i I(D_i = 0)$$

Generating posterior predictive samples and calculating deviance

The posterior predictive samples (PPS) and deviance of PPS was calculated using the following nimble function:

```
library(nimble)

ppSamplerNF <- nimbleFunction(
  setup = function(model, samples) {

    # theta
    theta <- colnames(samples)
```

```
    theta <- model$topologicallySortNodes(theta)

    # nodes to simulate
    simNodes <- model$getNodeNames()[!(model$getNodeNames() %in% theta)]

    # data nodes
    dataNodes <- model$getNodeNames(dataOnly = TRUE)

    n <- length(model$expandNodeNames(dataNodes, returnScalarComponents = TRUE))
    vars <- colnames(samples[, theta])

    # subset posterior samples to just theta (top nodes in graph)
    samples_sub <- samples[, theta]

  },
  run = function(samples = double(2)) {

    nSamp <- dim(samples)[1]
    ppSamples <- matrix(nrow = nSamp, ncol = n + 1)

    for(i in 1:nSamp) {

      # add theta to model
      values(model, vars) <<- samples_sub[i, ]

      # update nodes based on theta
      model$simulate(simNodes, includeData = TRUE)

      # save PPS
      ppSamples[i, 1:n] <- values(model, dataNodes)

      # store deviance of each sample
      ppSamples[i, n + 1] <- 2 * model$calculate(dataNodes)
    }
    returnType(double(2))
    return(ppSamples)
  })
```

The posterior predictive samples (PPS) and deviance were calculated and saved using the nimble function:

```
# generate posterior predictive sampler
ppSampler <- ppSamplerNF(
  myModel, # uncompiled model
  samples # posterior samples
)

# compile posterior predictive samples
cppSampler <- compileNimble(
  ppSampler,
  project = CmyModel # compiled model
)

# run compiled function
```

```
ppSamples_via_nf <- cppSampler$run(samples)
```

Calculating deviance of data

Bayesian p-values are calculated by comparing the deviance of the posterior predictive samples and the deviance of the observed data. Deviance of the data was calculated using the following nimble function:

```
calc_deviance_D <- nimbleFunction(
  setup = function(model, samples) {

    # theta = top nodes and latent states
    theta <- colnames(samples)

    # calculate model graph dependencies of theta to update
    deps <- model$getDependencies(theta, self = TRUE)

    # data nodes
    dataNodes <- model$getNodeNames(dataOnly = TRUE)

  },
  run = function(samples = double(2)) {

    nSamp <- dim(samples)[1]
    deviance <- rep(NA, nSamp)

    for(i in 1:nSamp) {

      # add theta
      values(model, theta) <<- samples[i, ]

      # update dependencies
      model$calculate(deps)

      # calculate deviance
      deviance[i] <- 2 * model$calculate(dataNodes)
    }
    returnType(double(1))
    return(deviance)
  })
```

The deviance of the data was calculated and saved using the nimble function:

```
# generate deviance calculator
devianceCalculator <- calc_deviance_y(
  myModel, # uncompiled model, contains data
  samples # posterior samples
)

# compile deviance calculator
CdevianceCalculator <- compileNimble(
  devianceCalculator,
  project = CmyModel # compiled model, contains data
)
```

```r
# run compiled function
data_deviance_via_nf <- CdevianceCalculator$run(samples)
```

Model checking with deviance

The Bayesian p-value calculated using deviance as the proportion of $T(D_i) > T(D_i^{\text{rep}})$. The deviance p-value is 0.43.

Figure A6.1: Histogram of deviance generated for each posterior sample for D (red) and D^{rep} (blue).

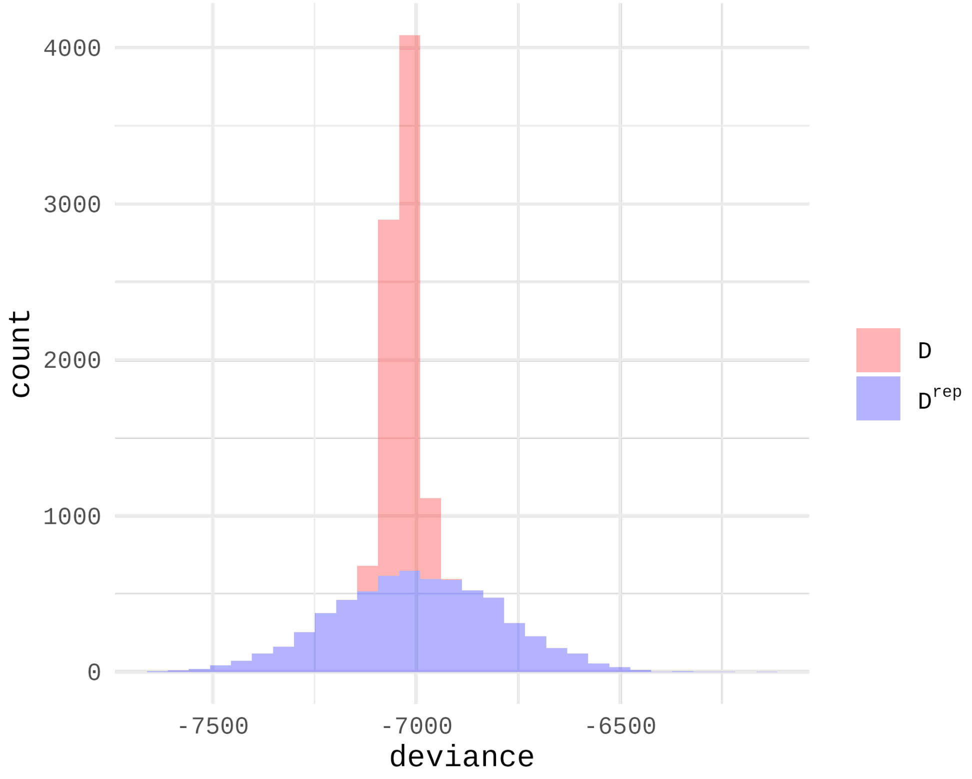

Model checked with zero inflation

The Bayesian p-value using the targeted zero-inflation check was calculated as the proportion of $T(D_i) > T(D_i^{\text{rep}})$, where D refers to the count data, $C(x)_{t,j,y}$. The zero-inflation check p-value is 0.95.

Figure A6.2: Histogram of proportion of zeros in each set, i, of posterior predictive samples, D^{rep}. Red line indicates the proportion of zeros in the count data, D.

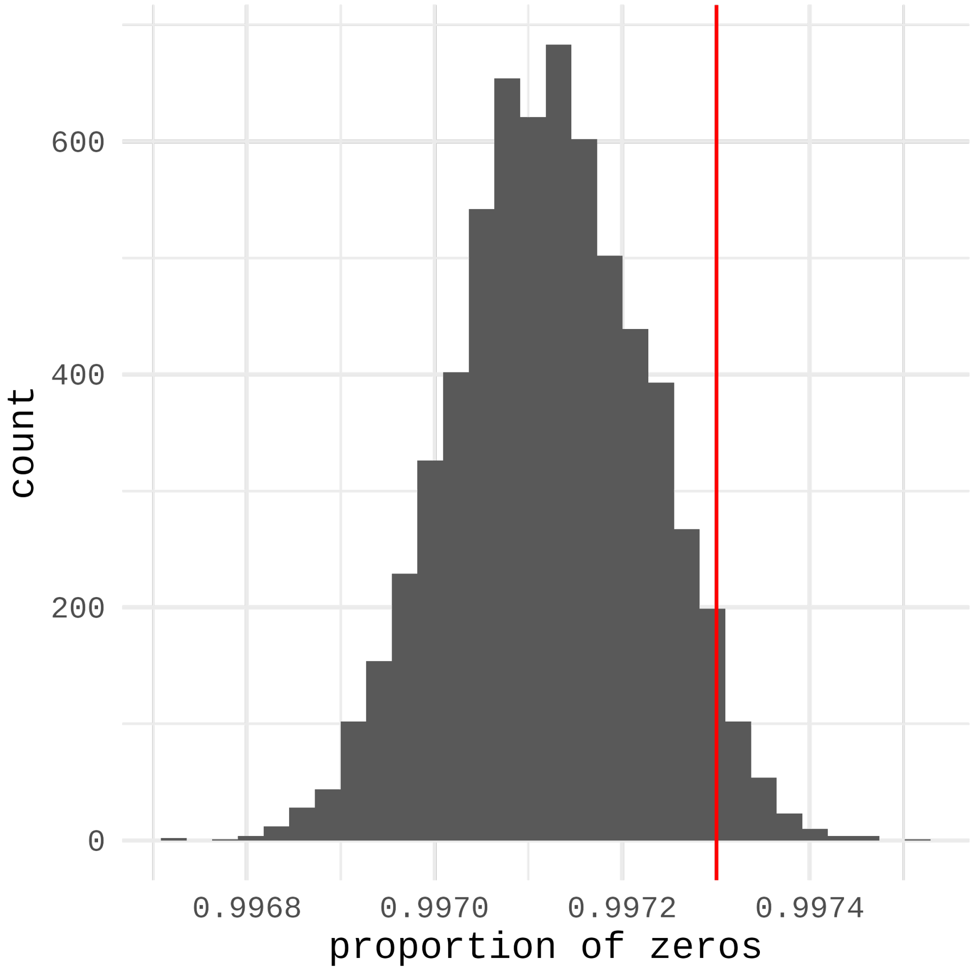

Appendix 6.3: Testing model assumptions with posterior predictive checks

Integrated population models assume that the same population process underlies all data sets in the combined model, and estimation of "additional", previously unidentifiable parameters is particularly sensitive to violations of IPM model assumptions. Knowing whether the same population process has generated multiple data sets can be challenging, but posterior predictive checks may be a reliable way of testing model assumptions.

The mark-recapture data set (D3) was critical for inferring the trap hazard rates. The model described in the main text was fit using data from Roche Cove on Vancouver Island in British Columbia, Canada. Roche Cove is similar to Drayton Harbor, where the multi-year time series data (D1) was collected. Both sites have shallow, open tide flats and creeks that drain in the bay with similar estuary mouth habitats.

Another available mark-recapture data set is from Seadrift Lagoon in California, USA, taken from Grosholz et al. (2021), where the co-authors conducted an extensive capture-mark-recapture experiment to estimate green crab population size (Grosholz et al. 2021). While the same Fukui traps were employed at Seadrift Lagoon as in Drayton Harbor and Roche Cove, the lagoon is small and man-made. The lagoon is so contained that both intra- and interspecies dynamics are likely different than expected in a more natural embayment. To test model assumptions and the sensitivity of our results to the choice of dataset, we fit the same model

described in the main text, while replacing the Roche Cove mark-recapture data (D3) with the Seadrift Lagoon mark-recapture data. More details on the data and updated model can be found below in Appendix 6.3.1.

We first compare the parameter estimates of the maximum trap hazard rates, $H_F^{\max}$, $H_M^{\max}$, $H_S^{\max}$, for Fukui, Minnow, and Shrimp traps, respectively, as well as the parameter estimates of green crab adult abundance in year 1, λ^A, and the recruit abundance for year i, λ_i^R. The estimates of the Fukui and Shrimp hazard rates are higher when the model is fitted with Seadrift Lagoon data, and the estimate of adult abundance in year 1 is lower when the model is fitted with Seadrift Lagoon data.

Table A6: Posterior summaries, including the mean and 95% credibility interval (highest density interval) with the model fit with the Roche Cove mark-recapture data (main text) and model fit with the Seadrift Lagoon mark-recapture data.

\

parameter	Roche Cove	Seadrift Lagoon
h_F^{max}	0.0001784 (0.0001498, 0.0002084)	0.0003012 (0.0002683, 0.0003351)
h_M^{max}	0.0003912 (0.0002752, 0.0005132)	0.0004025 (0.0002906, 0.0005248)
h_S^{max}	0.003937 (0.003186, 0.004752)	0.004593 (0.003848, 0.005352)
λ^A	441.3 (360, 529.1)	312.5 (266.6, 358.8)
λ_1^R	697.6 (322.1, 1172.1)	753 (341.9, 1263.7)
λ_2^R	50.32 (27.68, 75.8)	45.78 (26.85, 65.7)
λ_3^R	1394 (842, 2086)	1364 (842, 1981)
λ_4^R	61.35 (21.47, 109.49)	70.88 (26.37, 123.22)

We then generated posterior predictive samples using the Seadrift Lagoon posterior and calculated Bayesian p-values based on deviance and proportion of zeros as described above.

The Bayesian p-value calculated using deviance as the proportion of $T(D_i) > T(D_i^{\text{rep}})$. The deviance p-value is 0.71.

Figure A6.3: Histogram of deviance generated for each posterior sample for D (red) and D^{rep} (blue) with the Seadrift Lagoon data.

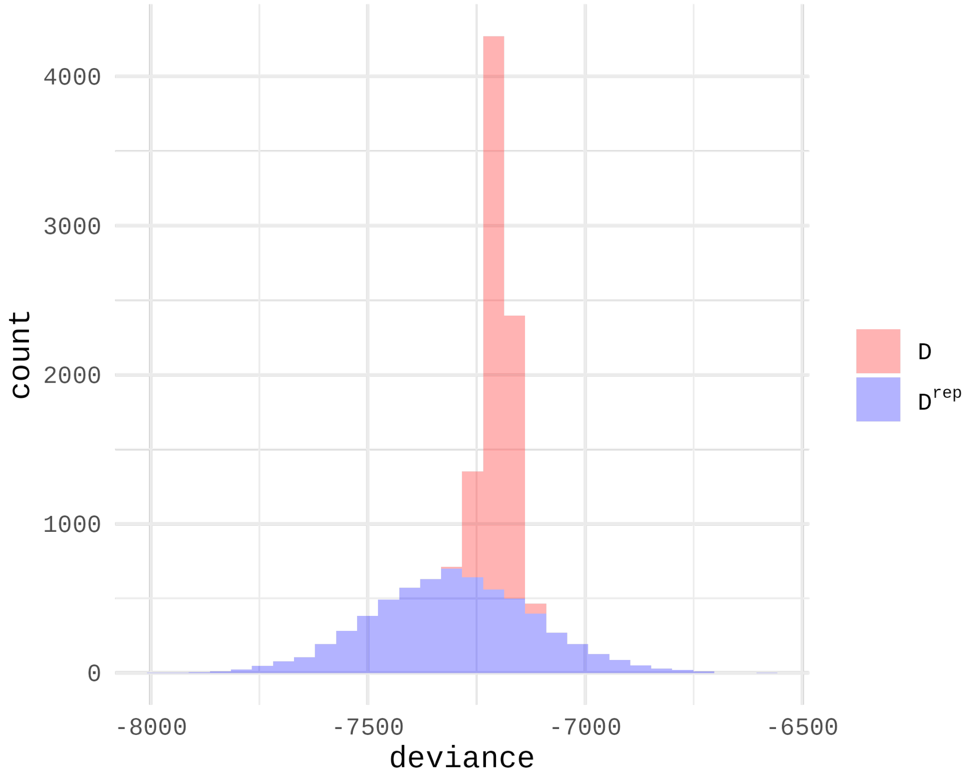

Model checked with zero inflation

The Bayesian p-value using the targeted zero-inflation check was calculated as the proportion of $T(D_i) > T(D_i^{\text{rep}})$, where D refers to the count data, $C(x)_{t,j,y}$. The zero-inflation check p-value is 0.99.

Figure A6.4: Histogram of proportion of zeros in each set, i, of posterior predictive samples, D^{rep}, calculated with the Seadrift Lagoon data. Red line indicates the proportion of zeros in the count data, D.

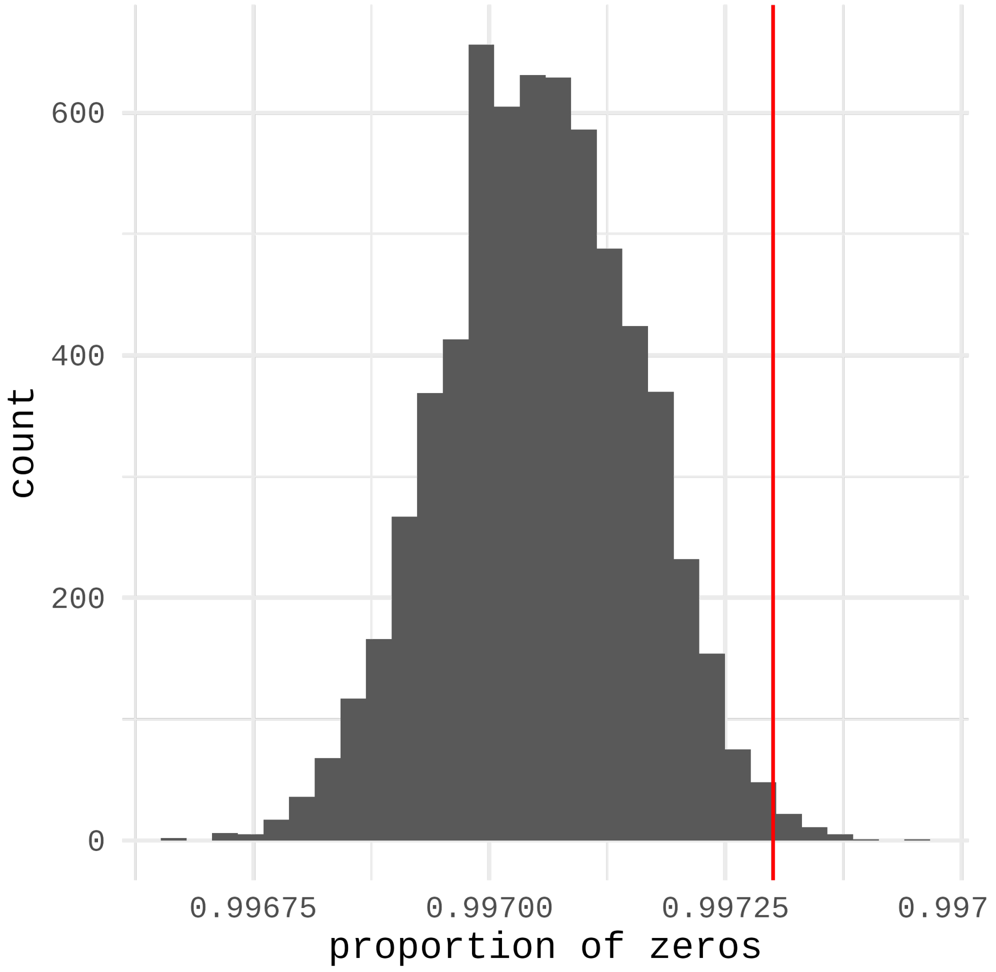

These Bayesian p-values are similar generated with the model fit with Roche Cove dataset (D3) and both suggest reasonable model fits. Although the inclusion of the Seadrift lagoon data did not qualitatively change the results, inference on some parameters, like $h_F^{\max}$, did change moderately quantitatively. While posterior predictive checks may be useful for broadly understanding if multiple datasets reflect the same data-generating process, selecting datasets for an integrated population model may be limited to judgemetns about habitat similarity.

Appendix 6.3.1: Seadrift Lagoon mark-recapture data

The authors in Grosholz et al., 2021 deployed 15 Fukui traps in June within the lagoon. Over the succeeding three days, they marked all crabs caught by clipping two spines on the carapace, recording their size, and releasing them back to their original location. Within successive weeks as part of an eradication program, crabs were removed, and the number and carapace width of retrieved (marked and unmarked) crabs were recorded.

Data was retrieved from NSF's Biological and Chemical Oceanography Data Management Office at https://www.bco-dmo.org/person/699768. While the mark-recapture experiment took place from 2011-2018, we only used data from one year, 2011, since the marking data was unambiguous.

The Seadritf Lagoon mark-recapture data consists of 1) the count of marked and released crabs of discrete size x_i in the first time period, $n^{\text{mc}}(x_i)$, and 2) the count of recaptured and marked crabs of discrete size x_i in the second time period, $m^{\text{mc}}(x_i)$. Here we describe how the mark-recapture observation process is formulated with Seadrift Lagoon data (i.e., Equations 23-26 in main text):

These mark-recapture data follow a binomial distribution:

$$m^{\text{mc}}(x_i) \sim \text{Binomial}(n_{t_2}^{\text{mc}}(x_i), p^{\text{mc}}(x_i))$$

where $p^{\text{mc}}(x_i)$ is the total probability of capture based on the total number of Fukui traps set, O^{mc}, over the time period Δb^{mc}:

$$p^{\text{mc}}(x) = 1 - \exp\left(-\sum_{j=1}^{O^{\text{mc}}} H_{F,j}(x)\Delta b_j^{\text{mc}}\right)$$

These data also informed components of the growth and natural mortality kernel (Table 1; Figure 2). The number of marked and released crabs, $n^{\text{mc}}(x_i)$, at t_1^{mc} underwent seasonal growth and natural mortality to the next time period, t_2^{mc}.

$$n_{t_2}^{\text{mc}}(x') = \int_{x\in\Omega} K^{\text{mc}}(x', x) n_{t_1}^{\text{mc}}(x) dx$$

References

Conn, Paul B, Devin S Johnson, Perry J Williams, Sharon R Melin, and Mevin B Hooten. 2018. "A Guide to Bayesian Model Checking for Ecologists." *Ecological Monographs* 88 (4): 526–42.

Grosholz, Edwin D, Gail Ashton, Marko Bradley, Chris Brown, Lina Ceballos-Osuna, Andrew Chang, Catherine de Rivera, et al. 2021. "Stage-Specific Overcompensation, the Hydra Effect, and the Failure to Eradicate an Invasive Predator." *Proceedings of the National Academy of Sciences* 118 (12): e2003955118.

King, Ruth, Byron Morgan, Olivier Gimenez, and Steve Brooks. 2009. *Bayesian Analysis for Population Ecology.* Chapman; Hall/CRC.

Lunn, David, Christopher Jackson, Nicky Best, Andrew Thomas, and David Spiegelhalter. 2013. "The BUGS Book." *A Practical Introduction to Bayesian Analysis, Chapman Hall, London.*
