# Supplementary material for: An integrated integral projection model (IPM2 ) to disentangle size‐structured harvest and natural mortality
Source: J Anim Ecol. 2025 Nov 11;95(1):157–74. doi: 10.1111/1365-2656.70176 (PMC12775557; doi:10.1111/1365-2656.70176)
Supplement: Supplementary file 7 — Figure S1. Growth kernel, Gtx′x (Equation 6), that describes the probability of transitioning from size x to size x′ at time t. Results show the probability of growing from x to x′ over a 2‐week period (i.e. t to t+1) when t corresponds to April 1st, July 1st, October 1st and January 1st. Red dashed line shows the line where x′=x. The growth kernel is calculated with the mean posterior estimates of growth parameters. Figure S2. Population forecasts in response to varying removal efforts, relative to no removal. Size distributions show the ratio of the equilibrium crab abundance in each size class, NEx, when no removal occurred (NExeffort=0) relative to a removal effort greater than zero (NExeffort>0). Ratios are calculated based on size‐structured abundance at the end of the year after overwinter mortality when 112 traps, 560 traps or 2800 traps were applied evenly over a trapping season of 14 biweeks with either A. Fukui traps, B. Minnow traps or C. Shrimp traps. A ratio of one means that the size‐structured abundance after no removal equals the size‐structured abundance after application of Z traps. A ratio less than one means that the application of Z traps removes decreases the size‐structured abundance, relative to no removal. A ratio greater than one means that the application of Z traps increases the size‐structured abundance, relative to no removal. Solid line indicates the median size‐structured abundance across simulation replicates, and the shaded area indicates ±1 standard deviation across simulation replicates. Table S1. Total equilibrium abundance across all size classes, NE for population forecasts in response to varying removal efforts. Mean refers to the mean NE across all simulation replicates, and sd refers to the standard deviation of NE across all simulation replicates. [file JANE-95-157-s001.docx]

# Supplemental Information

**Figure S1:** Growth kernel, $G_{t}\left( x',x \right)$ (Equation 6), that describes the probability of transitioning from size $x$ to size $x'$ at time $t$. Results show the probability of growing from $x$ to $x'$ over a two week period (i.e., $t$ to $t+1$) when $t$ corresponds to April 1st, July 1st, October 1st, and January 1st. Red dashed line shows the line where $x'=x$. The growth kernel is calculated with the mean posterior estimates of growth parameters.
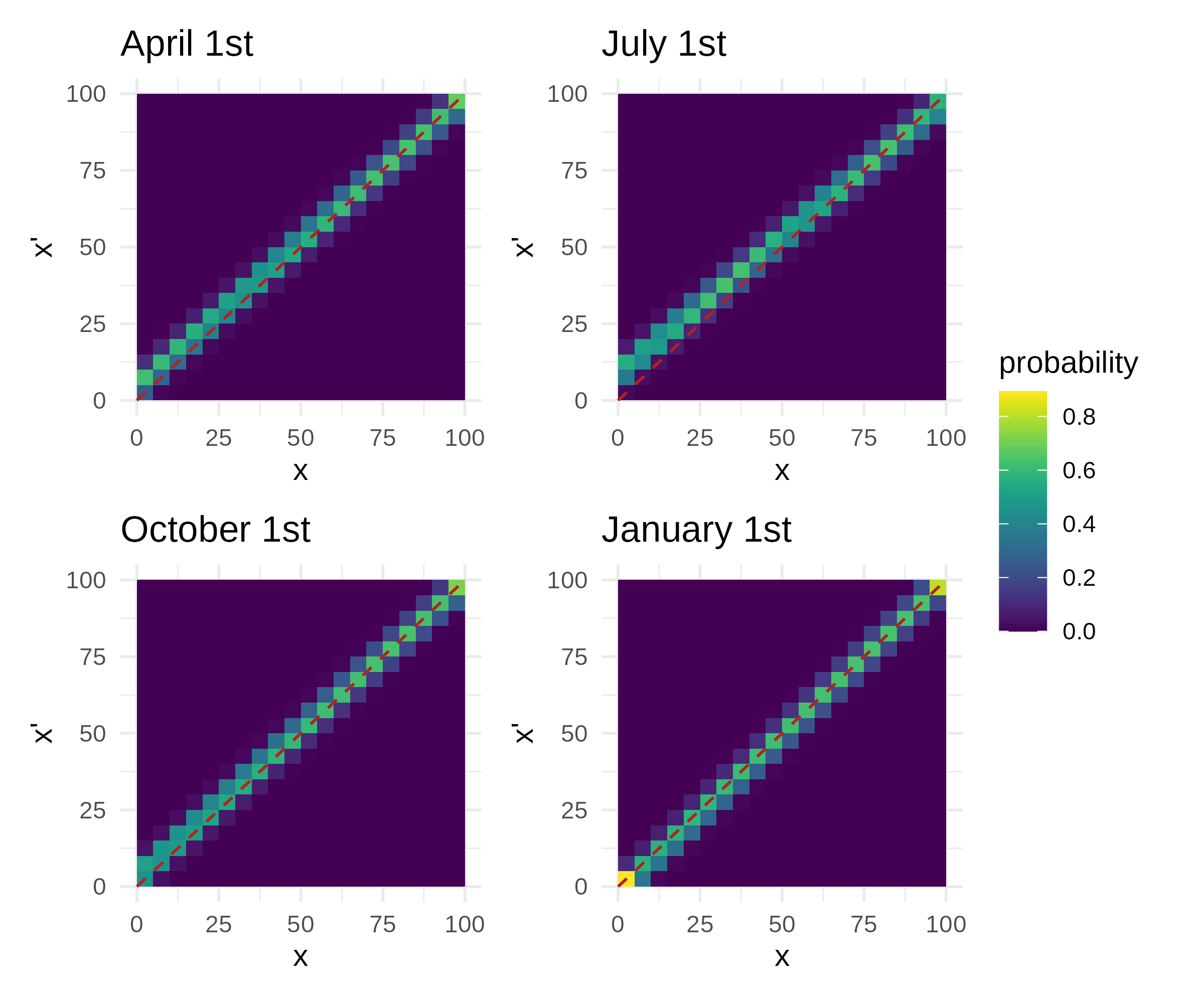


**Figure S2:** Population forecasts in response to varying removal efforts, relative to no removal. Size distributions show the ratio of the equilibrium crab abundance in each size class, $N^{E}\left( x \right)$, when no removal occurred ($N^{E}\left( x \right)_{\text{effort}=0}$) relative to a removal effort greater than zero ($N^{E}\left( x \right)_{\text{effort}>0}$). Ratios are calculated based on size-structured abundance at the end of the year after overwinter mortality when 112 traps, 560 traps, or 2800 traps were applied evenly over a trapping season of 14 biweeks with either *A.* Fukui traps, *B.* Minnow traps, or *C.* Shrimp traps. A ratio of one means that the size-structured abundance after no removal equals the size-structured abundance after application of Z traps. A ratio less than one means that the application of Z traps removes decreases the size-structured abundance, relative to no removal. A ratio greater than one means that the application of Z traps increases the size-structured abundance, relative to no removal. Solid line indicates the median size-structured abundance across simulation replicates, and the shaded area indicates $\pm1$ standard deviation across simulation replicates.
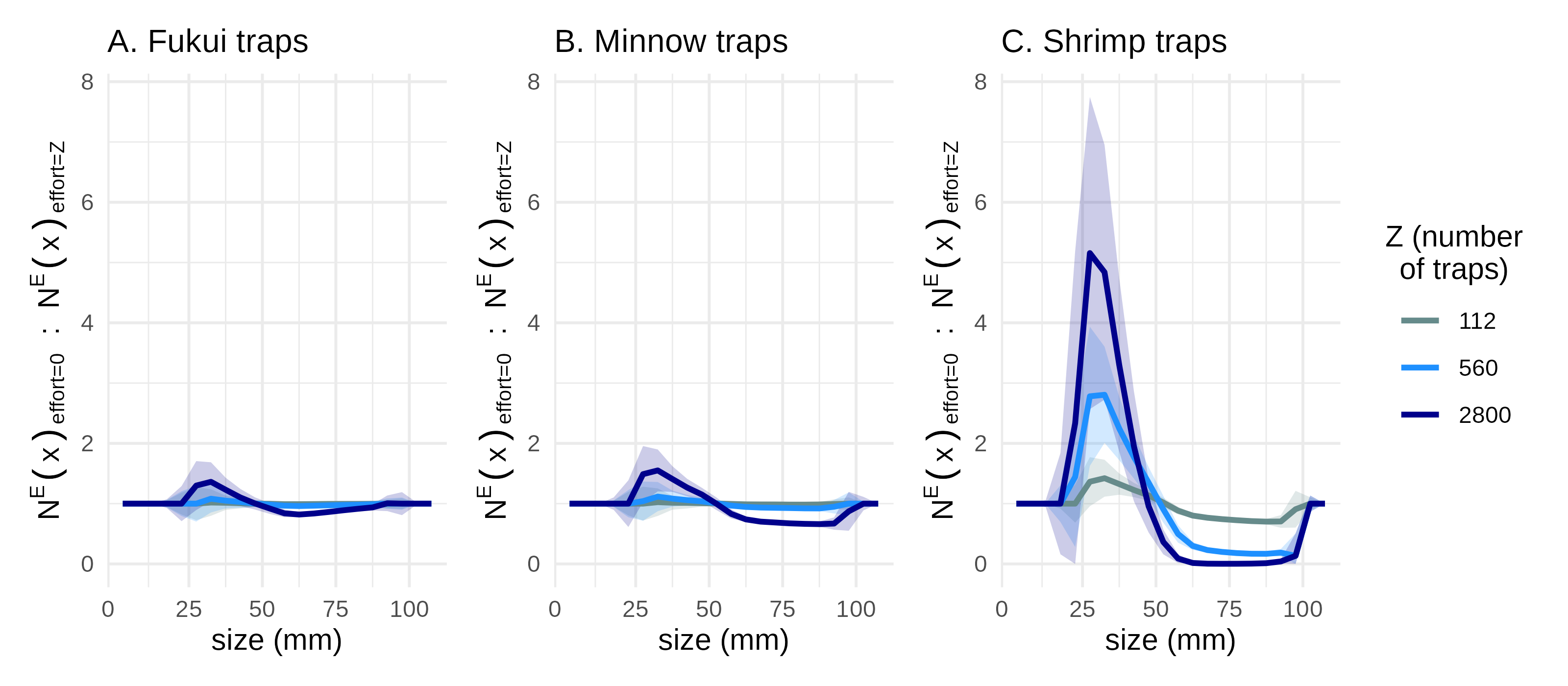


**Table S1**: Total equilibrium abundance across all size classes, $N^{E}$ for population forecasts in response to varying removal efforts. Mean refers to the mean $N^{E}$ across all simulation replicates, and sd refers to the standard deviation of $N^{E}$ across all simulation replicates.

| type | effort | $N^{E}$ (mean) | $N^{E}$ (sd) |
| --- | --- | --- | --- |
| NA | 0 | 268.5 | 66.5 |
| Fukui | 28 | 268.2 | 66.5 |
| Fukui | 112 | 267.4 | 66.2 |
| Fukui | 560 | 262.3 | 64.7 |
| Fukui | 840 | 259.3 | 63.8 |
| Fukui | 1400 | 253.2 | 62.1 |
| Fukui | 2800 | 238.6 | 58.3 |
| Minnow | 28 | 267.9 | 66.3 |
| Minnow | 112 | 265.9 | 65.8 |
| Minnow | 560 | 255.7 | 63 |
| Minnow | 840 | 249.4 | 61.2 |
| Minnow | 1400 | 237.3 | 58.1 |
| Minnow | 2800 | 209.3 | 51.5 |
| Shrimp | 28 | 255.9 | 63.2 |
| Shrimp | 112 | 221.2 | 54.7 |
| Shrimp | 560 | 117.1 | 38.3 |
| Shrimp | 840 | 92.9 | 36 |
| Shrimp | 1400 | 74.5 | 33.6 |
| Shrimp | 2800 | 62.1 | 29.6 |
